# Alternative covariance structures in mixed-effects models: Addressing intra- and inter-individual heterogeneity

**DOI:** 10.3758/s13428-023-02133-1

**Published:** 2023-05-25

**Authors:** Shelley A. Blozis, Madeline Craft

**Affiliations:** Department of Psychology, University of California, Davis, Davis, California USA

**Keywords:** Autocorrelation, Procedural learning, Response latency, Working memory, Mixed-effects location scale models, Nonlinear mixed-effects models

## Abstract

Mixed-effects models for repeated measures and longitudinal data include random coefficients that are unique to the individual, and thus permit subject-specific growth trajectories, as well as direct study of how the coefficients of a growth function vary as a function of covariates. Although applications of these models often assume homogeneity of the within-subject residual variance that characterizes within-person variation after accounting for systematic change and the variances of the random coefficients of a growth model that quantify individual differences in aspects of change, alternative covariance structures can be considered. These include allowing for serial correlations between the within-subject residuals to account for dependencies in data that remain after fitting a particular growth model or specifying the within-subject residual variance to be a function of covariates or a random subject effect to address between-subject heterogeneity due to unmeasured influences. Further, the variances of the random coefficients can be functions of covariates to relax the assumption that these variances are constant across subjects and to allow for the study of determinants of these sources of variation. In this paper, we consider combinations of these structures that permit flexibility in how mixed-effects models are specified to understand within- and between-subject variation in repeated measures and longitudinal data. Data from three learning studies are analyzed using these different specifications of mixed-effects models.

**Supplementary Information:**

The online version contains supplementary material available at 10.3758/s13428-023-02133-1.

Repeated measures and longitudinal data are essential in studies of growth or change in human behavior. Data collected across repeated occasions, whether the occasions are closely spaced (e.g., studies of human learning or ecological momentary assessments to understand variation in mood) or span across a relatively long period of time (e.g., longitudinal studies of human growth and development), are necessary to understand if, when, or how behaviors change and how other variables might influence the process.

Mixed-effects models are widely applied for the analysis of repeated measures and longitudinal data. These models emphasize the individual by specifying a growth model at the subject level and provide a framework to study within- and between-subject variation in measured behaviors. In specifying a model, a function, typically assumed to be common to all individuals, is selected to describe the response, but one or more of the function coefficients are subject-specific to allow for individual differences in certain aspects of change or development. In this way, the subject-specific model can be used to account for variation in scores within individuals, and the subject-specific coefficients permit the study of how individuals differ in aspects of change. An appealing quality of these models is that the coefficients of a function can vary according to covariates at the subject level to study how specific aspects of change or development depend on covariates.

Careful selection of a growth function that effectively summarizes a large collection of data by a relatively small number of parameters can aid in the analysis and interpretation of repeated measures and longitudinal data, especially when the form of change is complex (Cudeck & Harring, 2007). Further, if the chosen function is effective in capturing variation within individuals, then the occasion-specific residuals will be independent within subjects. In other words, it is typically reasonable to assume that scores within subjects are correlated, and a mixed-effects model accounts for these correlations with the random coefficients of the growth model. If, however, a function is ineffective in fully accounting for this variation, some dependencies between residuals will remain.

Previous work has documented the impact on model fit and statistical inference of poorly specified residual covariance structures at the first level of a mixed-effects model. It is well known that applications of mixed-effects models that erroneously assume that the residuals are independent with constant variance across occasions have consequences for the estimates of the variances of the random coefficients of the subject-level model (Baek et al., 2020; Baek & Ferron, 2020; Blozis & Harring, 2021; Chi & Reinsel, 1989; Ferron et al., 2002; Harring & Blozis, 2014; Joo et al., 2019; Sivo et al., 2005). Thus, in fitting a mixed-effects model, the analyst faces a need to balance parsimony and interpretability in selecting a growth model with the need to adequately address dependencies of scores within subjects.

A special class of mixed-effects models, known as mixed-effects location scale models, have expanded the ways in which to model intra- and inter-individual differences by including submodels for the variance of the residuals at the first level of the model, as well as for the variances of the random coefficients at the second level (for three-level models, see Lin et al., 2018; Nestler et al., 2018). Importantly, these extensions provide a framework to relax assumptions of homogeneity of the covariance structure at each model level, in addition to providing a means to study the determinants of these sources of variation in data. These models grew from the need to model repeated-measures data that generally did not involve growth or change, and the residuals at level 1 were assumed to be independent (see Hedeker et al., 2008), but their application to growth and change processes (e.g., McNeish, 2021; Williams et al., 2019) have not consistently incorporated this earlier literature that has emphasized the importance of considering alternative residual covariance structures when the assumption of independence between the level-1 residuals is not tenable. Shown to be useful in applications of linear growth models (Nestler, 2021, 2022), this paper joins these two methodological areas within the context of fitting nonlinear mixed-effects models, thus broadening their use in problems involving relatively complex forms of change.

To accomplish this, data from three learning experiments are utilized to motivate researchers to expand their thinking about how they might formulate the covariance structure of a mixed-effects model to study within- and between-individual differences in repeated measures that follow complex forms of change. We first develop a general framework to increase options in how one specifies the covariance structure of a mixed-effects model with special attention to how covariates may be incorporated into the structure. The examples are then presented. We develop syntax for using maximum likelihood (ML) estimation of models using SAS PROC NLMIXED by expanding on the developments of Harring and Blozis (2014). A discussion follows with implications and directions for future research.

## Mixed-effects models for repeated measures and longitudinal data

Resources on mixed-effects models are numerous. Among the many published materials on mixed-effects models are books that serve a range of purposes, with some providing a general resource (Wu, 2010) or focus on a particular software program (Rabe-Hesketh & Skrondal, 2012) or area of application (Brown & Prescott, 2015), and others emphasizing specific topics, such as nonlinear mixed-effects models (Fiedler-Kelly & Owen, 2014) or autoregressive mixed-effects models (Funatogawa & Funatogawa, 2018). We assume readers are familiar with common formulations of a mixed-effects model, including linear and nonlinear models. We give a brief description of the model that serves as a starting point for our developments here.

Let *y*_ij_ be the observed measure for individual *i* at time *j*, where *i* = 1,..., *N* and *j* = 1,..., *n*_i_, letting *N* denote the number of subjects and *n*_i_ the number of measures for individual *i*. Let *t*_ij_ be the time when *y*_ij_ was observed. A mixed-effects model for *y*_ij_ is

1 $${y}_{\mathrm{ij}}=f\left({t}_{\mathrm{ij}},{\mathbf{X}}_{\mathrm{ij}},{{\boldsymbol{\uptheta}}}_{\mathrm{i}},{\mathbf{W}}_{\mathrm{i}},{\boldsymbol{\upgamma}}\right)+{\varepsilon }_{\mathrm{ij}}$$

where $f\left(\cdot \right)$ is a function (e.g., linear, quadratic, logistic) assumed to characterize the growth or developmental trend of the response. In (1), ${y}_{\mathrm{ij}}$ is a function of* t*_ij_, a set of covariates ${\mathbf{X}}_{\mathrm{ij}}$ (that usually includes 1 for the intercept of the model) that vary with ${y}_{\mathrm{ij}}$, a set of subject-specific coefficients ${{\boldsymbol{\uptheta}}}_{\mathrm{i}}$ that link ${\mathbf{X}}_{\mathrm{ij}}$ and ${y}_{\mathrm{ij}}$, a set of between-subject covariates ${\mathbf{W}}_{\mathrm{i}}$, a set of fixed coefficients ${\boldsymbol{\upgamma}}$ that link ${\mathbf{W}}_{\mathrm{i}}$ and ${y}_{\mathrm{ij}}$, and a time- and subject-specific residual ${\varepsilon }_{\mathrm{ij}}$. Coefficients in ${{\boldsymbol{\uptheta}}}_{\mathrm{i}}$ can be fixed, random, or the sum of a fixed and a random effect. That is, for the q^th^ coefficient in ${{\boldsymbol{\uptheta}}}_{\mathrm{i}}$, ${\theta }_{qi}={\alpha }_{q}+{u}_{qi}$, where ${\alpha }_{q}$ is the fixed effect and ${u}_{qi}$ is the corresponding random effect. The random effects are assumed to be independently and identically distributed (i.i.d.) as normal across subjects with mean **0** and covariance matrix ${\boldsymbol{\Phi}}$, where the dimensions of ${\boldsymbol{\Phi}}$ depend on the number of random effects in ${{\boldsymbol{\uptheta}}}_{\mathrm{i}}$. The residual ${\varepsilon }_{\mathrm{ij}}$ is the response not accounted for by the subject-specific model given by $f\left({t}_{\mathrm{ij}},{\mathbf{X}}_{\mathrm{ij}},{{\boldsymbol{\uptheta}}}_{\mathrm{i}},{\mathbf{W}}_{\mathrm{i}},{\boldsymbol{\upgamma}}\right)$. The set of residuals **ε**_i_ = (${\varepsilon }_{i1}$,...,${\varepsilon }_{i{n}_{\mathrm{i}}}$)′ is assumed to be i.i.d. normal across subjects with mean **0** and covariance matrix **Θ**_ε_, where the dimensions of **Θ**_ε_ depends on *n*_i_.

### Within-subject covariance structure

The residual covariance matrix characterizes the variation and between-occasion covariation of the deviations of observations from their expected values. For a growth model that fully accounts for the within-subject dependencies of scores between occasions and given that the residual variances are equal across time, a simple structure for the covariance matrix is appropriate: ${{\boldsymbol{\Theta}}}_{\varepsilon }={\sigma }_{\varepsilon }^{2}{\mathbf{I}}_{{n}_{i}}$. Other options ought to be considered, however, if within-subject dependencies between scores remain, even after accounting for covariates, or if the variances of the residuals are not equal across occasions. The assumption of homogeneity of the residual covariance structure between subjects can be relaxed by specifying a model in which the covariance structure differs between groups, such as by allowing the variance of a simple covariance structure to differ between groups: ${{\boldsymbol{\Theta}}}_{\varepsilon k}={\sigma }_{\varepsilon k}^{2}{\mathbf{I}}_{{n}_{ik}}$, where *k* denotes group membership. The variance itself could also be a function of measured covariates (Stroup et al., 2018), similar to linear regression models that use an exponential function to model the residual variance to address heterogeneity (Aitkin, 1987; Cook & Weisberg, 1983; Harvey, 1976; Carroll & Ruppert, 1988). In such cases, between-subject heterogeneity of the residual variance is assumed to be due to measured covariates.

An alternative is a mixed-effects location scale model in which the residual variance is assumed to be due to unmeasured covariates, in addition to possibly being due to measured covariates, by including a random subject effect in a model for the residual variance (Hedeker et al., 2008; Hedeker & Nordgren, 2013). For example, in a model that assumes independent residuals between occasions, an exponential function (that ensures the result is positive) can be used to model the residual variance (cf. Hedeker et al., 2008):

$${\sigma }_{i}^{2}=exp\left({\tau }_{0}+{v}_{i}\right)$$

where the exponentiated value of ${\tau }_{0}$ is the within-person variance for a person whose random effect ${v}_{i}$ is equal to 0. The random effect ${v}_{i}$ is assumed to be i.i.d. lognormal across subjects. Similar to earlier work (e.g., Harvey, 1976), the model for the residual variance can be expanded to include measured covariates. For example, let ${X}_{ij}$ and ${W}_{i}$ denote a time-varying covariate and a between-subjects covariate, respectively, a model for ${\sigma }_{ij}^{2}$ that is assumed to vary by individual and according to the two covariates is

$$exp\left({\tau }_{0}+{{\tau }_{1}{X}_{ij}+{\tau }_{2}{W}_{i}+v}_{i}\right),$$

where ${\tau }_{0}$, when exponentiated, is the within-person variance for a person whose random effect ${v}_{i}$, within-subject covariate ${X}_{ij}$, and between-subject covariate ${W}_{i}$ are equal to 0. The coefficients ${\tau }_{1}$ and ${\tau }_{2}$ are the effects of the within- and between-subject covariates, respectively, on the residual variance, with a positive effect indicating that a higher level of a covariate corresponds to greater within-subject variation and a negative effect indicating that a higher level of a covariate corresponds to a lower degree of within-subject variation. For example, using a mixed-effects location scale model, Blozis et al. (2020) reported greater between-subject heterogeneity of the within-subject variance of daily time spent engaged in leisure activities on weekends versus weekdays and for women versus men. The random effect ${v}_{i}$ is the residual from the regression and is assumed to be independent and lognormally distributed between subjects. Importantly, the variance of the random scale effect ${v}_{i}$, if different from zero, reflects an additional source of between-subject variation due to unobserved sources, conditional on the observed covariates.

As mentioned previously, earlier reports have documented the impact of mis-specifying the level-1 residual covariance structure of a mixed-effects model, including reports of relatively poor model fit, and perhaps more importantly, consequences for the estimated variances of the random subject effects at the second level. Ignoring correlations between residuals at the occasion level has, for example, been shown to result in overestimation of the variances of the random effects at the second level of a linear mixed-effects model (Chi & Reinsel, 1989; Ferron et al., 2002; Sivo et al., 2005). Thus, the connection between how the occasion-level covariance structure is specified and its impact on the subject-level covariance structure deserves attention when fitting a mixed-effects model to understand between-subject differences in a behavior studied over time. This point may be of particular importance when considering more advanced versions of a mixed-effects model, namely a mixed-effects location scale model. The implication is that ignoring serial correlations between the residuals at the occasion level may have consequences for the estimated variances of the random effects at the subject level, including the between-subject random scale variance that is a key component of a mixed-effects location scale model.

### Between-subject covariance structure

Mixed-effects models are subject-specific models because one or more of the coefficients of a growth function are assumed to vary between subjects. The between-subject covariance matrix of the random coefficients characterizes the degree to which individuals differ in the coefficients, as well as the extent to which the coefficients covary with one another. A useful aspect of a mixed-effects model is that it is possible to test if the random coefficients are related to subject-level covariates. This could be done to test if features of a growth or developmental process are associated with individual difference measures. If subject-level covariates are included in the regression equation of a random effect, the residual of that equation is a conditional random effect, and the variance of the conditional random effect represents variation in the random effect left unaccounted for.

As stated earlier, the variance of a random coefficient may be studied as a function of occasion- and subject-level covariates (Hedeker & Nordgren, 2013). In this way, it is possible to study the determinants of the variances of the random coefficients. To illustrate this, let ${\phi }_{0}^{2}$ denote the variance of a random intercept that is modeled as a function of an occasion- and a subject-level covariate. Using an exponential function to model the variance (cf. Hedeker & Nordgren, 2013),

$${\phi }_{0}^{2}=exp\left({\alpha }_{00}+{\alpha }_{01}{X}_{ij}+{\alpha }_{02}{W}_{i}\right)$$

where the exponentiated value of ${\alpha }_{00}$ is the variance of the random intercept when the covariates ${X}_{ij}$ and ${W}_{i}$ are equal to 0, and ${\alpha }_{01}$ and ${\alpha }_{02}$ are the effects of ${X}_{ij}$ and ${W}_{i}$, respectively, on the variance. A positive covariate effect would indicate greater between-subject variation in the random intercept with an increase in the covariate, and a negative effect would indicate a decrease in between-subject variation with an increase in the covariate. In a study of positive affect in adolescent cigarette smokers, for example, Hedeker et al. (2008) reported greater between-subject variation in a random intercept for individuals identified as loners relative to others and less variation among novelty seekers and 10th grade students relative to others.

### Estimation

Estimation of linear mixed-effects models can be conducted using statistical software programs that use methods applicable to the estimation of other linear multivariate models, such as linear structural equation models with latent variables (Blozis, 2007). In a mixed-effects location scale model, the variances at the occasion level and the variances of the random coefficients at the subject level are typically expressed using exponential functions, and thus involve the estimation of nonlinear parameters, and more generally, estimation of a nonlinear mixed-effects model. Among the software packages developed for maximum likelihood (ML) estimation of nonlinear mixed-effects models, SAS PROC NLMIXED (Wolfinger, 1999) has been widely used to estimate mixed-effects location scale models. PROC NLMIXED is well suited for the estimation of these models because the procedure makes it convenient to include the nonlinear models for the variance of a random effect at the subject level and the variance of the residual at the occasion level to model heterogeneity of variance at both levels (Hedeker et al., 2008).

PROC NLMIXED is also adaptable for ML estimation of the mixed-effects location scale models developed here in which the residual covariance structures do not conform to the procedure's default model specifications. Specifically, we consider models in which the residual covariance structure assumes an first-order autoregressive (AR(1)) structure to help address the possibility of correlation between adjacent residuals at the first level of a model, a possible indication that the explanatory portion of a growth model does not fully account for the within-subject variation. The default residual covariance structure in PROC NLMIXED is one in which the residuals are assumed to be independent between occasions with constant variance across occasions and subjects. The GENERAL model statement option permits a user-defined likelihood function of a given model. Using this option, it is possible to specify alternative residual covariance structures, such as an AR(1) structure with a fixed variance and autocorrelation coefficient (Harring & Blozis, 2014) or a structure with a random scale and autocorrelation as discussed in this paper.

PROC NLMIXED offers ML estimation using Gaussian quadrature and a dual quasi-Newton optimization routine (SAS Institute Inc., 2015). To fit the models presented in this paper, we relied on guidance provided by Kiernan et al. (2012) by providing reasonable starting values. For each of the data sets, we started by fitting a fixed-effects model to obtain estimates of the fixed effects of the growth curve and the effects of the covariates on growth parameters. We then built up models by adding random effects, one at a time, and updating the starting values for each model based on estimates obtained for previous models. Syntax for fitting models to the flight simulation data (the second example) is in the Appendix. Tests were carried out using a type 1 error rate of .05.

In the examples that follow, SAS PROC NLMIXED was used for estimation. The default method of estimation in this procedure involves approximating the marginal loglikelihood using an adaptive Gaussian quadrature method (Pinheiro & Bates, 1995). In a given problem, a good approximation requires an adequate number of quadrature points and appropriate centering and scaling of the abscissas for the random effects. The adaptive method has the advantage of requiring fewer quadrature points and has been shown to perform well when good starting values are provided and the number of random effects is not large (Pinheiro & Bates, 1995). For some of the models considered here, estimation using adaptive Gaussian quadrature (including the Laplace approximation) stopped with a report that no valid parameter points were found. We instead used Gaussian–Hermite quadrature that approximates each integral by a weighted average of the integrand that is evaluated at specific points over a grid centered at 0. Following guidance by Carlin et al. (2001) to use a high number of quadrature points when using nonadaptive Gaussian quadrature, models were estimated using 30 quadrature points. Although an increase in the number of grid points can increase the precision of the approximation of the integral, an increase can result in a computationally intensive analysis. We observed increasing stability in the parameter estimates as the number of quadrature points was increased, but recommend caution when fitting such complex methods using this method of estimation.

## Examples

The first of three data sets is analyzed using models that test possible autocorrelation and between-subject heterogeneity of the residual covariance structure at the first level of a model. This first example is one in which covariates are not available for study; thus the example illustrates the utility of considering alternative residual covariance structures at the first level of the model. The second and third data sets include individual difference measures that are studied in relation to learning. These two examples include individual difference measures that are used to study how such measures may serve as determinants of the different aspects of variation in responses at the subject level.

Each of the three examples involves response data that tend to follow nonlinear trends. One of the appealing aspects of fitting a growth model to repeated measures is that a large number of data values per subject may be summarized using a function that is parameterized by a relatively small number of coefficients. With the addition of random coefficients, the corresponding covariance structure imposed by a mixed-effects model is a parsimonious representation of the variances and covariances of the data. It may not be reasonable, however, to assume that conditional on the subject-specific growth model that the residuals are independent. That is, although a given growth model may do well in summarizing trends in the individual-level responses, it may not be reasonable to assume that it completely addresses within-subject dependencies, especially given that a function based on a small number of parameters is used to summarize a relatively large number of data points. With a need to balance parsimony and interpretability in selecting a growth model, it may be reasonable to consider alternative covariance structures at the first level of the model with the sensible acknowledgement that the imposed structure, including AR(1), is not assumed to be the specific structure that generated the dependencies in the data, but rather, that the AR(1) process may help to better represent the correlation structure of the data.

### Example 1: Performance on a complex procedural learning task

**Fig. 1:**
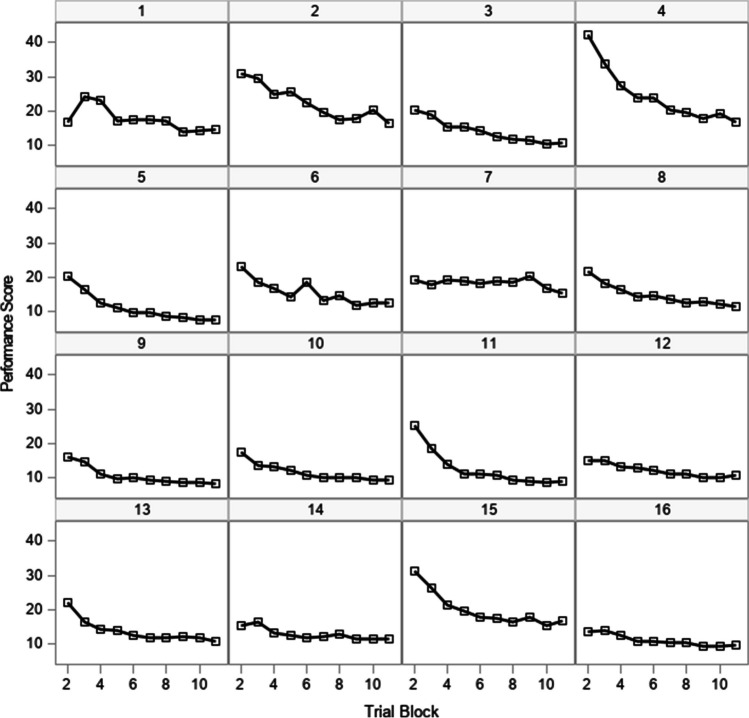
Response latencies on a procedural learning task for a selection of 16 participants

Data from a procedural learning task described in Woltz (1988) represent response latencies for 393 participants on 11 learning trial blocks (64 trials per block) (henceforth referred to as “trials”) that occurred in one session. Data from the first trial are excluded from analysis because it is assumed that these scores reflect participants’ adjustments to the task. Supplemental Table S1 gives descriptive statistics for trials 2–11. A plot of scores for 16 selected participants in Fig. 1 suggests that response latencies decrease at a nonconstant rate as trials progress, with scores leveling off towards the latter part of the session. We fit a series of models to these scores while making different assumptions about the residual covariance structure at the trial level, including the addition of an AR(1) structure and heterogeneity of the residual variance.

Analysis of the first data set began by fitting an exponential and a logistic growth function to the scores to test which best accounted for the data while assuming that the trial-level residuals were independent, with constant variance across trials and subjects. Each function included three subject-specific parameters representing the initial level, a lower asymptote, and a learning rate parameter. Responses were positively skewed within trials due to a subset of participants having relatively long response times. Given this, each function was applied to the data assuming that scores followed one of three response distributions— normal, gamma, or lognormal—with the latter two being continuous and positively skewed distributions. Based on the Akaike information criterion (AIC) and Bayesian information criterion (BIC)^1^ fit indices, the exponential growth function that assumed that the residuals were lognormal provided the best fit. Using log-transformed (base 10) scores (assumed to be normally distributed), this model was provisionally taken as the best fitting and used to test different assumptions about the residual covariance structure.

The exponential growth model for response ${y}_{\mathrm{ij}}$ was specified as

2 $${y}_{\mathrm{ij}}={\beta }_{1i}-\left({\beta }_{1i}-{\beta }_{0i}\right)exp\left\{-{\beta }_{2i}{t}_{ij}\right\}+{\varepsilon }_{\mathrm{ij}},$$

where ${t}_{ij}$ denotes the *j*^th^ trial for subject *i*. For subject *i*, ${\beta }_{0i}$ is the performance score at the first trial, ${\beta }_{1i}$ is the potential performance level (lower asymptote), and ${\beta }_{2i}$ is the rate parameter that combines with *t*_ij_ to represent the learning rate. At the subject level, each coefficient was a function of a fixed and random effect:

3a $${\beta }_{0i}={\gamma }_{00}+{u}_{0i},$$

3b $${\beta }_{1i}={\gamma }_{10}+{u}_{1i},$$

3c $${\beta }_{2i}={\gamma }_{20}+{u}_{2i},$$

where ${\gamma }_{00}$, ${\gamma }_{10}$, and ${\gamma }_{20}$ are the response level at the first trial, the potential performance level, and the rate parameter, respectively, for a subject whose random effects are equal to 0.

Next, we fit two sets of models using the exponential growth function in (2). These are summarized here, followed by more detailed descriptions. Models in the first set were mixed-effects models and those in the second were mixed-effects location scale models. The first model in each set assumed that the residuals were independent between trials, and the second in each set assumed that the residuals were correlated between trials. All three growth coefficients in the first and second models of each set were random, as in (3a)–(3c). The third model in each set assumed that the residuals were correlated between trials but that the rate parameter of the growth model was fixed. By fitting these particular models, we could test whether the growth function could be simplified in terms of the number of random coefficients in exchange for using an AR(1) structure (cf. Chi & Reinsel, 1989) and whether there was evidence of heterogeneity of the residual variance across subjects. We next provide greater detail about these models.

In the first set of models, Model 1a assumed that the residuals in (2) were i.i.d. normal and independent between trials with constant variance across the 10 trials: ${{\boldsymbol{\Theta}}}_{\varepsilon }={\sigma }_{\varepsilon }^{2}{\mathbf{I}}_{10}$, where ${\sigma }_{\varepsilon }^{2}$ is the common variance. The covariance matrix of the random effects at the subject level was assumed to be homogeneous across subjects and specified as

4 $${\boldsymbol{\Phi}}=\left[\begin{array}{ccc}{\phi }_{{u}_{0}}^{2}& & \\ {\phi }_{{u}_{1}{u}_{0}}& {\phi }_{{u}_{1}}^{2}& \\ {\phi }_{{u}_{2}{u}_{0}}& {\phi }_{{u}_{2}{u}_{1}}& {\phi }_{{u}_{2}}^{2}\end{array}\right],$$

where ${\phi }_{{u}_{0}}^{2}$, ${\phi }_{{u}_{1}}^{2}$, and ${\phi }_{{u}_{2}}^{2}$ are the variances of the random intercept, asymptote, and rate parameter, respectively, and ${\phi }_{{u}_{1}{u}_{0}}$, ${\phi }_{{u}_{2}{u}_{0}}$, and ${\phi }_{{u}_{2}{u}_{1}}$ are their covariances.

In Model 1b, the residual covariance matrix was assumed to follow an AR(1) structure:

5 $${{\boldsymbol{\Theta}}}_{\varepsilon }={\sigma }_{\varepsilon }^{2}\left[\begin{array}{ccccc}1& & & & \\ \rho & 1& & & \\ {\rho }^{2}& \rho & 1& & \\ \vdots & \vdots & \vdots & \ddots & \\ {\rho }^{9}& {\rho }^{8}& {\rho }^{7}& \cdots & 1\end{array}\right],$$

where ${\sigma }_{\varepsilon }^{2}$ is a common variance assumed to be constant across trials and subjects and $\rho$ is a fixed autocorrelation coefficient. The covariance matrix of the random effects of the growth model was assumed to have the same structure as in (4). Model 1c assumed the AR(1) residual structure in (5). The growth model assumed a random intercept and asymptote and a fixed rate parameter, thus reducing the dimensions of ${\boldsymbol{\Phi}}$:

$${\boldsymbol{\Phi}}=\left[\begin{array}{cc}{\phi }_{{u}_{0}}^{2}& \\ {\phi }_{{u}_{1}{u}_{0}}& {\phi }_{{u}_{1}}^{2}\end{array}\right].$$

The next set of models were mixed-effects location scale models. In Model 2a, the residuals were assumed to be independent between trials with constant variance across trials and between-subject heterogeneity of the residual variance: ${{\boldsymbol{\Theta}}}_{{\varepsilon }_{i}}={\sigma }_{{\varepsilon }_{i}}^{2}{\mathbf{I}}_{10}$, where

6 $${\sigma }_{{\varepsilon }_{i}}^{2}=exp\left({\tau }_{0}{+v}_{i}\right).$$

The exponentiated value of ${\tau }_{0}$ is the residual variance for a subject whose random effect ${v}_{i}$ is equal to 0. The random scale effect ${v}_{i}$ accounts for heterogeneity of variance due to unmeasured sources and is assumed to be lognormally and independently distributed between subjects. Given the random scale effect in (6), the covariance matrix of the random effects at the subject level includes the variance of the random scale effect and its covariances with the random growth coefficients:

7 $${\boldsymbol{\Phi}}=\left[\begin{array}{cccc}{\phi }_{{u}_{0}}^{2}& & & \\ {\phi }_{{u}_{1}{u}_{0}}& {\phi }_{{u}_{1}}^{2}& & \\ {\phi }_{{u}_{2}{u}_{0}}& {\phi }_{{u}_{2}{u}_{1}}& {\phi }_{{u}_{2}}^{2}& \\ {\phi }_{{vu}_{0}}& {\phi }_{v{u}_{1}}& {\phi }_{v{u}_{2}}& {\phi }_{v}^{2}\end{array}\right],$$

where ${\phi }_{v}^{2}$ is the variance of the random scale effect. Its covariances with the random growth coefficients are the first three elements in the last row of the matrix.

In Model 2b, the covariance matrix of the residuals followed an AR(1) structure for which the residual variance could vary by subject:

8 $${{\boldsymbol{\Theta}}}_{{\varepsilon }_{ij}}={\sigma }_{{\varepsilon }_{ij}}^{2}\left[\begin{array}{ccccc}1& & & & \\ \rho & 1& & & \\ {\rho }^{2}& \rho & 1& & \\ \vdots & \vdots & \vdots & \ddots & \\ {\rho }^{9}& {\rho }^{8}& {\rho }^{7}& \cdots & 1\end{array}\right],$$

where ${\sigma }_{{\varepsilon }_{ij}}^{2}=exp\left({\tau }_{0}{+v}_{i}\right)$. The autocorrelation coefficient was assumed to be constant across subjects. In Model 2c, an AR(1) structure as specified for Model 2b was assumed, and the rate parameter of the growth model was assumed to be fixed across subjects. The mean and covariance structures of Models 1a–1c and 2a–2c are summarized in Supplemental Table S4.

## Results

Point estimates and 95% confidence intervals (CI) for Models 1a–1c and 2a–2c are in Table 1. When estimating these models, the variances at both levels were expressed by exponential functions, and so the calculated variances are provided in the lower part of the table. Models are first compared in terms of fit, and then conclusions are drawn. We first compared Model 1a that assumed independence between the residuals and Model 1b that assumed an AR(1) structure. A deviance test comparing the models was significant (χ^2^(1 *df*) =148, *p* < .001), suggesting that dependencies in scores were not entirely accounted for by the growth function, and indeed, the estimated autocorrelation was .38. This result is consistent with a preference for Model 1b according to the AIC and BIC indices, where both indices are lower under Model 1b. We next compared the fit of Models 1a and 1c to test whether the number of random growth coefficients could be reduced in exchange for using an AR(1) structure (cf. Chi & Reinsel, 1989). That is, Model 1a is relatively complex due to the inclusion of three random growth coefficients, whereas Model 1c involves only two random growth coefficients and uses the AR(1) to help account for the dependencies of scores within subjects. Although the fit was better under Model 1c, suggesting that an AR(1) structure might be used to reduce the dimensionality of the model in terms of the number of random effects, the fit of Model 1b was best among the three, suggesting that the most complex model was preferred overall. Thus, assuming homogeneity of the residual variance across subjects, individuals differed in the three aspects of performance, but the subject-specific growth model did not capture all of the within-subject dependencies in scores.

**Table 1:** ML estimates of an exponential growth model for response latencies on a procedural learning task (n = 393)

	Mixed-effects models			Mixed-effects location scale models		
	Model 1a	Model 1b	Model 1c	Model 2a	Model 2b	Model 2c
Within-subject covariance structure	${\sigma }_{e}^{2}{\mathbf{I}}_{10}$	$AR(1)$ with ${\sigma }_{e}^{2}$	$AR(1)$ with ${\sigma }_{e}^{2}$	${\sigma }_{{e}_{i}}^{2}{\mathbf{I}}_{10}$	$AR\left(1\right)$ with ${\sigma }_{{e}_{i}}^{2}$	${AR(1)}_{i}$ with ${\sigma }_{{e}_{i}}^{2}$
Fixed effects	MLE [95% CI]	MLE [95% CI]	MLE [95% CI]	MLE [95% CI]	MLE [95% CI]	MLE [95% CI]
Initial level, ${\gamma }_{00}$	2.94 [2.92, 2.97]	2.93 [2.90, 2.96]	2.93 [2.90, 2.96]	2.94 [2.93, 2.95]	2.94 [2.92, 2.96]	2.94 [2.92, 2.96]
Asymptote, ${\gamma }_{10}$	2.30 [2.28, 2.32]	2.28 [2.25, 2.31]	2.36 [2.33, 2.38]	2.30 [2.28, 2.31]	2.31 [2.28, 2.34]	2.37 [2.35, 2.39]
Rate, ${\gamma }_{20}$	0.28 [0.26, 0.30]	0.24 [0.21, 0.27]	0.26 [0.24, 0.28]	0.25 [0.24, 0.26]	0.25 [0.23, 0.27]	0.28 [0.26, 0.30]
Within-subject covariance parameters						
${\tau }_{0}$	−10.0 [−10.1, −9.9]	−4.71 [−4.81, −4.61]	−4.59 [−4.68, −4.49]	−10.8 [−11.0, −10.6]	−5.10 [−5.22, −4.98]	−4.92 [−5.03, −4.80]
$\rho$		.38	.43		.34	.43
Between-subject covariance parameters						
Intercept, ${\alpha }_{00}$	−2.56 [−2.67, −2.45]	−2.56 [−2.72, −2.41]	−2.57 [−2.72, −2.42]	−2.76 [−2.85, −2.68]	−2.61 [−2.74, −2.49]	−2.65 [−2.78, −2.52]
Asymptote, ${\alpha }_{10}$	−3.15 [−3.25,−3.05]	−3.52 [−3.86, −3.18]	−3.17 [−3.33, −3.00]	−3.49 [−3.62, −3.37]	−3.60 [−3.84, −3.35]	−3.28 [−3.41, −3.15]
Rate, ${\alpha }_{20}$	−4.00 [−4.18,−3.82]	−4.29 [−4.54, −4.04]		−4.28 [−4.43, −4.14]	−4.38 [−4.58, −4.19]	
Scale, ${\alpha }_{v}$				0.97 [0.78, 1.15]	−0.52 [−0.71, −0.32]	−0.59 [−0.79, −0.40]
Corr(${u}_{1},{u}_{0}$), ${\phi }_{{u}_{1}{u}_{0}}$	.25	.60	.46	.42	.58	.52
Corr(${u}_{2},{u}_{0}$), ${\phi }_{{u}_{2}{u}_{0}}$	−.13	.17		.11	.11	
Corr(${u}_{2},{u}_{1}$), ${\phi }_{{u}_{2}{u}_{1}}$	.59	.35		.14	.06	
Corr(${u}_{v},{u}_{0}$), ${\phi }_{v{u}_{0}}$				.14	.23	.28
Corr(${u}_{v},{u}_{1}$), ${\phi }_{v{u}_{1}}$				.40	.48	.64
Corr(${u}_{v},{u}_{2}$), ${\phi }_{v{u}_{2}}$				−.47	−.46	
Additional variance estimates						
Residual, ${\sigma }_{{e}_{0}}^{2}$	4.4E−05	0.01	0.01	2.1E−05	0.01	
Initial, ${\phi }_{{u}_{1}}^{2}$	0.08	0.08	0.08	0.06	0.07	0.07
Asymptote, ${\phi }_{{u}_{2}}^{2}$	0.04	0.03	0.04	0.03	0.03	0.04
Rate, ${\phi }_{{u}_{3}}^{2}$	0.02	0.01		0.01	0.01	
Scale, ${\phi }_{v}^{2}$				2.63	0.60	0.55
−2lnL	−5792	−5940	−5871	−6595	−6727	−6641
AIC	−5771	−5917	−5855	−6565	−6696	−6618
BIC	−5732	−5874	−5824	−6511	−6638	−6575

Latency scores are log-transformed to base 10. The within-subject covariance structures are as follows: ${\sigma }_{e}^{2}{\mathbf{I}}_{10}$ denotes independence between trials and homogeneity of variance across trials and subjects; $AR(1)$ with ${\sigma }_{e}^{2}$ denotes a first-order autocorrelation with constant variance across trials and subjects; ${\sigma }_{{e}_{i}}^{2}{\mathbf{I}}_{10}$ denotes independence between trials, homogeneity of variance across trials and between-subject heterogeneity of variance; $AR(1)$ with ${\sigma }_{{e}_{i}}^{2}$ denotes a first-order autocorrelation with constant variance across trial blocks and between-subject heterogeneity of variance

Whereas Models 1a–1c are mixed-effects models that assumed homogeneity of the residual variance, Models 2a–2c are mixed-effects location scale models that permit between-subject heterogeneity of the residual variance. We compared the first model of the two sets, Models 1a and 2a, which differ in that the former assumed homogeneity of the residual variance and the latter assumed between-subject heterogeneity. The deviance test^2^ was significant (χ^2^(4 *df*) = 803, *p* < .001), suggesting between-subject heterogeneity of the residual variance. Deviance tests between Models 1b and 2b and between 1c and 2c (not reported here) result in comparable conclusions about the need to permit heterogeneity of the residual variance.

We next compared the fit of Models 2a and 2c to test whether the number of random growth coefficients could be reduced in exchange for using an AR(1) structure. Similar to comparisons between models in the first set, model fit according to the AIC and BIC values was better under Model 2c relative to 2a, suggesting again that an AR(1) structure might be used to reduce the dimensionality of the model in terms of the number of random effects. The fit of Model 2b, however, was the best (according to the AIC and BIC values) among the three, suggesting that the more complex model was preferred. Our overall conclusion about this performance measure is that there is evidence of individual differences in the three aspects of performance, but the subject-specific growth model did not fully capture the within-subject dependencies in scores, and there was significant within-subject variation about the subject-specific performance trajectories.

For these data there were slight differences in the estimated variances of the random growth coefficients between the models that allowed for autocorrelation between the level-1 residuals versus assuming independence and whether the model assumed heterogeneity of the residual variance or not (see Table 1). More remarkable, however, was the difference in the estimated variance of the random scale effect, a value that indicates the extent to which individuals differ from each other with regard to the variance of the residuals about the subject-specific trajectories. The estimated variance of the random scale effect was reduced from 2.63 under Model 2a that assumed independence of residuals between trials to 0.60 under Model 2b that assumed an AR(1) structure, suggesting the importance of considering an AR(1) structure when drawing inferences about the degree of between-subject heterogeneity of the residual variance. This result suggests that between-subject heterogeneity of the residual variance was due in part to dependencies between the residuals after accounting for systematic growth by the subject-specific model, again illustrating the importance of accounting for autocorrelation in a growth process.

### Example 2: Performance on a flight controller simulation task

The second data set is from a study of motivation, cognitive abilities, and skill acquisition (Kanfer & Ackerman, 1989). For 140 participants, the set includes repeated measures on a flight controller simulation task and a battery of motivation and cognitive ability measures. The task was designed to measure skill acquisition during a 100-minute period. The repeated measures are the number of planes brought in safely every ten minutes. Scores for the first trial are excluded from analysis, as it is assumed that participants used the first trial to adjust to the task. Scores for trials 2–10 are analyzed here. Two individual difference measures, mathematics knowledge (MK) and coding speed (CS), are used to study how each is related to performance. The MK score reflects general mathematical knowledge, including algebra and geometry. CS measures processing speed and accuracy by having subjects relate numbers in a list to information provided in a graph.

**Fig. 2:**
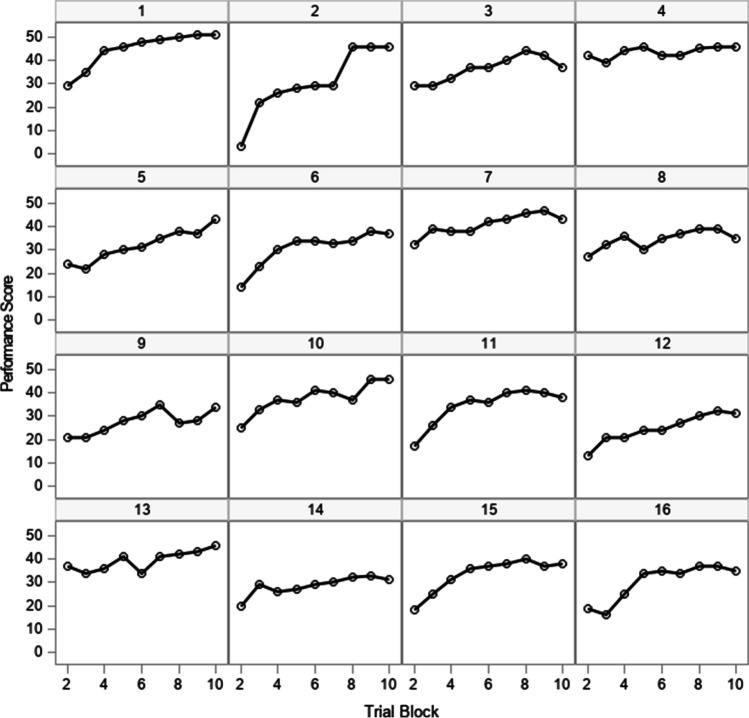
Performance scores on a flight simulation task for a selection of 16 participants

Descriptive statistics for the performance scores and individual difference measures are in Supplemental Table S2. The performance scores for 16 selected participants are displayed in Fig. 2. In Harring and Blozis (2014), this set of scores was analyzed, and among the models tested, the best fitting was one that assumed scores changed according to a negatively accelerated logistic function and the level 1 residuals followed an AR(1) structure, with the covariance structured assumed to be homogeneous across participants. Thus, from their analysis, the logistic function did not fully account for dependencies in the data, and as they showed, it was important to address this in the residual covariance structure by allowing scores between trials to correlate. This was especially important when drawing inferences about the variances of the random effects at the subject level because those estimates were impacted by the assumptions made about the residuals at the first level.

For these data, we combine a mixed-effects model that includes an AR(1) structure with features of a mixed-effects location scale model. Specifically, we use the same logistic function in Harring and Blozis (2014) and add the between-subject measures, MK and CS, to test their effects on specific aspects of learning. We relax the assumption of homogeneity of the residual covariance structure across subjects by including a random effect for the scale and autocorrelation coefficient, thus permitting the within-subject variance of the residuals and the autocorrelation coefficient to differ between subjects. Additionally, the variances of the random coefficients are studied as functions of the individual difference measures to test if the variances of the random growth coefficients are related to either of these measures.

A logistic growth function that included a random intercept ${\beta }_{0i}$, upper asymptote ${\beta }_{1i}$ and rate parameter ${\beta }_{2i}$ was applied to the performance measures:

9 $${y}_{\mathrm{ij}}=\frac{{\beta }_{0i}{\beta }_{1i}}{{\beta }_{0i}+\left({\beta }_{1i}-{\beta }_{0i}\right)exp\left\{-{\beta }_{2i}\left({t}_{ij}-1\right)\right\}}+{\varepsilon }_{\mathrm{ij}}.$$

For subject *i*, ${\beta }_{0i}$ is the performance score at the first trial, ${\beta }_{1i}$ is the potential performance level (upper asymptote), and ${\beta }_{2i}$ is the rate parameter that, when combined with *t*_ij_, governs the learning rate across trials. These coefficients were modeled as functions of MK and CS^3^:

10a $${\beta }_{0i}={\gamma }_{00}+{\gamma }_{01}{MK}_{i}+{\gamma }_{02}{CS}_{i}+{u}_{0i},$$

10b $${\beta }_{1i}={\gamma }_{10}+{\gamma }_{11}{MK}_{i}+{\gamma }_{12}{CS}_{i}+{u}_{1i},$$

10c $${\beta }_{2i}={\gamma }_{20}+{\gamma }_{21}{MK}_{i}+{\gamma }_{22}{CS}_{i}+{u}_{2i},$$

where ${\gamma }_{00}$, ${\gamma }_{10}$, and ${\gamma }_{20}$ are the performance levels at the first trial, the potential level, and the rate parameter for a subject with MK and CS scores equal to their respective sample means. The coefficients ${\gamma }_{01}$, ${\gamma }_{11}$, and ${\gamma }_{21}$ are the effects of MK, holding constant the effects of CS, on the three learning coefficients, respectively. The coefficients ${\gamma }_{02}$, ${\gamma }_{12}$, and ${\gamma }_{22}$ are the effects of CS, holding constant the effects of MK, on the three learning coefficients, respectively. The residuals of (10a)–(10c), ${u}_{0i}$, ${u}_{1i}$, and ${u}_{2i}$, are the respective subject-specific effects that remain after accounting for the effects of MK and CS.

Four models were applied to the data using the growth function in (9) with the level-2 equations in (10a)–(10c), with models differing according to the assumed covariance structure. The first two, Models 3a and 3b, were mixed-effects models that assumed homogeneity of the level 1 and level 2 covariance structures across subjects but differed in that Model 3a assumed that the residuals at the trial level were independent and Model 3b assumed that the residuals were correlated between trials (also see Harring & Blozis, 2014). The second two, Models 4a and 4b, were mixed-effects location scale models that assumed between-subject heterogeneity of the level 1 and level 2 covariance structures and differed in that Model 4a assumed that the residuals at the trial level were independent and Model 4b assumed that the residuals were correlated between trials. More details are given next.

Model 3a assumed that the residuals in (9) were i.i.d. normal and independent with constant variance across trials and subjects: ${{\boldsymbol{\Theta}}}_{\varepsilon }={\sigma }_{\varepsilon }^{2}{\mathbf{I}}_{9}$, where ${\sigma }_{\varepsilon }^{2}$ is the common variance. The covariance matrix of the three conditional random effects in (10a)–(10c) was assumed to be homogeneous across subjects, similar to (4) in the first example. In Model 3b, the residual covariance matrix at level 1 was assumed to follow an AR(1) structure similar to (5), assuming homogeneity of the residual variance and autocorrelation coefficient. Similar to Model 3a, the covariance matrix of the conditional random effects in (10a)–(10c) was assumed to be homogeneous across subjects.

Model 4a was mixed-effects location scale model in which the level 1 residuals were assumed to be independent between trials with constant variance, but the variance could vary between subjects according to the measured covariates MK and CS and unmeasured sources: ${{\boldsymbol{\Theta}}}_{{\varepsilon }_{ij}}={\sigma }_{{\varepsilon }_{i}}^{2}{\mathbf{I}}_{9}$, where

$${\sigma }_{{\varepsilon }_{i}}^{2}=exp\left({\tau }_{0}+{{\tau }_{1}{MK}_{i}+{{\tau }_{2}CS}_{i}+v}_{i}\right),$$

where ${\tau }_{0}$, when exponentiated, is the residual variance for a subject whose MK and CS scores are each at their respective sample mean, and ${v}_{i}$ is equal to 0; ${\tau }_{1}$ and ${\tau }_{2}$ are the effects of MK and CS, respectively, on the exponent with each adjusted for the effect of the other. The random scale effect ${v}_{i}$ is the residual that remains after accounting for the two measured variables and was assumed to be lognormally and independently distributed between subjects. Given the added random scale effect, the level 2 covariance matrix included the variance of the random scale and its covariances with the conditional random effects of the growth model, like that shown in (7).

Model 4b was a mixed-effects location scale model that assumed correlated residuals at the trial level (similar to Model 3b) but allowed for between-subject heterogeneity of the covariance structures at levels 1 and 2. Specifically, the covariance matrix of the residuals at level 1 was assumed to follow a random AR(1) structure:

$${{\boldsymbol{\Theta}}}_{{\varepsilon }_{i}}={\sigma }_{{\varepsilon }_{i}}^{2}\left[\begin{array}{ccccc}1& & & & \\ {\rho }_{i}& 1& & & \\ {\rho }_{i}^{2}& {\rho }_{i}& 1& & \\ \vdots & \vdots & \vdots & \ddots & \\ {\rho }_{i}^{8}& {\rho }_{i}^{7}& {\rho }_{i}^{6}& \cdots & 1\end{array}\right],$$

where

$$\begin{array}{c}{\sigma }_{{\varepsilon }_{i}}^{2}=exp\left({\tau }_{0}+{{\tau }_{1}{MK}_{i}+{{\tau }_{2}CS}_{i}+v}_{i}\right),\\ {\rho }_{i}=\rho +{w}_{i},\end{array}$$

where the interpretation of the model for the within-subject variance ${\sigma }_{{\varepsilon }_{i}}^{2}$ was identical to that for Model 4a. The coefficient $\rho$ is the autocorrelation coefficient for a subject whose random effect ${w}_{i}$ is equal to 0; ${w}_{i}$ is a random effect for the autocorrelation coefficient assumed to be approximated by a normal distribution. Given the random scale and random autocorrelation coefficient (${v}_{i}$ and ${w}_{i}$) of the level 1 covariance structure and the three conditional random effects of the growth model (${u}_{0i}$, ${u}_{1i}$ and ${u}_{2i}$ from 10a to 10c), the covariance matrix at level 2 is as shown in (11) where, with the exception that the variance of each conditional random effect of the growth model is a function of the covariates, MK and CS:

11 $$\Phi = \left[\begin{array}{ccccc} \phi^{2}_{u_{0}}&&&&\\\phi_{u_{1}u_{0}}&\phi^{2}_{u_{1}}&&&\\\phi_{u_{2}u_{0}}&\phi_{u_{2}u_{1}}&\phi^{2}_{u_{2}}&&\\\phi_{vu_{0}}&\phi_{vu_{1}}&\phi_{vu_{2}}&\phi^{2}_{v}&\\\phi_{wu_{0}}&\phi_{wu_{1}}&\phi_{wu_{2}}&\phi_{wv}&\phi^{2}_{w}\end{array}\right]$$

$$\begin{array}{c}{\phi }_{{u}_{0}}^{2}=exp\left\{{\alpha }_{00}+{\alpha }_{01}{MK}_{i}+{\alpha }_{02}{CS}_{i}\right\},\\ {\phi }_{{u}_{1}}^{2}=exp\left\{{\alpha }_{10}+{\alpha }_{11}{MK}_{i}+{\alpha }_{12}{CS}_{i}\right\},\\ {\phi }_{{u}_{2}}^{2}=exp\left\{{\alpha }_{20}+{\alpha }_{21}{MK}_{i}+{\alpha }_{22}{CS}_{i}\right\}.\end{array}$$

The mean and covariance structures of Models 3a, 3b, 4a, and 4b are summarized in Table S1.

## Results

The most complex of the four models was Model 4b. This model included a random scale effect and a random autocorrelation coefficient, thus allowing subjects to differ in the degree of variation of their residuals about their fitted trajectories and in the degree of autocorrelation between their residuals, respectively. For these data, the estimated variance of the random effect for the autocorrelation was very close to 0. From this, we concluded that subjects did not vary significantly in the autocorrelation parameter, and so Model 4b was simplified by assuming a fixed autocorrelation coefficient. Reports on Model 4b henceforth relate to this simplified model. The estimates and 95% confidence intervals from the models are given in Table 2. As variances at both levels of each model were expressed using exponential functions, Table 2 includes the calculated variances in the lower part of the table.

**Table 2:** ML estimates of a logistic growth model for flight simulation performance scores (n = 140)

	Mixed-effects model		Mixed-effects location scale model	
	Model 3a	Model 3b	Model 4a	Model 4b
Within-subject covariance structure	${\sigma }_{e}^{2}{\mathbf{I}}_{9}$	$AR(1)$ with ${\sigma }_{e}^{2}$	${\sigma }_{{e}_{i}}^{2}{\mathbf{I}}_{9}$	$AR(1)$ with ${\sigma }_{{e}_{i}}^{2}$
Fixed effects	MLE [95% CI]	MLE[95% CI]	MLE[95% CI]	MLE[95% CI]
Initial level, ${\gamma }_{10}$	18.4 [16.9, 19.8]	15.3 [14.1, 16.6]	16.2 [15.8, 16.7]	17.1 [16.1, 18.0]
MK, ${\gamma }_{11}$	1.26 [1.16, 1.36]	1.48 [1.35, 1.60]	1.59 [1.53, 1.65]	1.86 [1.69, 2.03]
CS, ${\gamma }_{12}$	−0.26 [−0.28, −0.23]	−0.30 [−0.34, −0.26]	−0.22 [−0.26, −0.20]	−0.19 [−0.30, −0.09]
Asymptote, ${\gamma }_{20}$	39.5 [38.5, 40.5]	37.9 [36.4, 39.5]	39.1 [38.2, 39.9]	39.1 [37.6, 40.5]
MK, ${\gamma }_{21}$	0.59 [0.39, 0.79]	0.62 [0.29, 0.94]	0.69 [0.51, 0.87]	0.65 [0.30, 0.99]
CS, ${\gamma }_{22}$	0.08 [0.02, 0.15]	0.05 [−0.07, 0.17]	0.06 [0.01, 0.11]	0.09 [−0.01, 0.19]
Rate, ${\gamma }_{30}$	0.69 [0.62, 0.77]	0.74 [0.65, 0.82]	0.72 [0.67, 0.78]	0.69 [0.59, 0.78]
MK, ${\gamma }_{31}$	−0.04 [−0.04, −0.03]	−0.04 [−0.05, −0.03]	−0.05 [−0.06, −0.04]	−0.04 [−0.06, −0.03]
CS, ${\gamma }_{32}$	0.003 [0.002, 0.005]	0.004 [−0.002, 0.01]	0.003 [0.0002, 0.01]	0.001 [−0.005, 0.007]
Within-subject covariance parameters				
${\tau }_{0}$	2.22 [2.13,2.31]	2.55 [2.35, 2.74]	2.11 [1.94, 2.27]	2.54 [2.28, 2.79]
MK, ${\tau }_{1}$			−0.04 [−0.07, −0.002]	−0.03 [−0.06, 0.005]
CS, ${\tau }_{2}$			0.01 [−0.004, 0.02]	0.01 [−0.01, 0.02]
$\rho$		.39		.44
Between-subject covariance parameters				
Intercept, ${\alpha }_{10}$	4.52 [4.37,4.67]	4.92 [4.75, 5.09]	4.50 [4.44, 4.55]	4.19 [4.05, 4.32]
MK, ${\alpha }_{11}$			−0.08 [−0.09, −0.07]	−0.12 [−0.16, −0.08]
CS, ${\alpha }_{11}$			0.02[0.02, 0.03]	0.03[0.01, 0.04]
Asymptote, ${\alpha }_{20}$	4.22 [3.94,4.50]	4.12 [3.73, 4.50]	4.13 [3.94, 4.32]	3.85 [3.51, 4.20]
MK, ${\alpha }_{21}$			−0.003 [−0.04, 0.04]	0.02 [−0.05, 0.08]
CS, ${\alpha }_{11}$			0.01 [−0.01, 0.02]	0.02 [−0.01, 0.05]
Rate, ${\alpha }_{30}$	−2.33 [−2.56,−2.10]	−2.27 [−2.69, −1.86]	−2.10 [−2.29, −1.91]	−2.79 [−3.36, −2.14]
MK, ${\alpha }_{31}$			−0.15 [−0.18, −0.13]	−0.25 [−0.35, −0.15]
CS, ${\alpha }_{11}$			−0.004 [−0.01, 0.01]	0.01 [−0.01, 0.04]
Scale, ${\alpha }_{v}$			−0.67 [−1.07, −0.26]	−1.13 [−1.64, −0.62]
Corr(${u}_{2},{u}_{1}$), ${\phi }_{{u}_{2}{u}_{1}}$	.58	.67	.68	.59
Corr(${u}_{3},{u}_{1}$), ${\phi }_{{u}_{3}{u}_{1}}$	−.56	−.63	−.60	−.46
Corr(${u}_{3},{u}_{2}$), ${\phi }_{{u}_{3}{u}_{2}}$	−.51	−.55	−.43	−.39
Corr(*v* $,{u}_{1}$), ${\phi }_{v{u}_{1}}$			−.32	−.31
Corr(*v* $,{u}_{2}$), ${\phi }_{v{u}_{2}}$			−.14	−.07
Corr(*v* $,{u}_{3}$), ${\phi }_{v{u}_{3}}$			.19	.28
Additional variance estimates				
Residual, ${\sigma }_{{e}_{0}}^{2}$	9.21	12.7	7.9	12.7
Initial, ${\phi }_{{u}_{1}}^{2}$	91.7	137.	89.7	65.7
Asymptote, ${\phi }_{{u}_{2}}^{2}$	68.0	61.5	62.3	47.1
Rate, ${\phi }_{{u}_{3}}^{2}$	0.10	0.10	0.12	0.06
Scale, ${\phi }_{v}^{2}$			0.51	0.32
−2lnL	7346.3	7329.9	7291.8	7264.0
AIC	7382.7	7369.0	7362.5	7337.9
BIC	7425.4	7414.0	7430.2	7407.4

MK = Math Knowledge, CS = Coding Speed (each centered to their respective sample mean). The within-subject covariance structures are as follows: ${\sigma }_{e}^{2}{\mathbf{I}}_{9}$ denotes independence between trials and homogeneity of variance across trial blocks and subjects; $AR(1)$ with ${\sigma }_{e}^{2}$ denotes a first-order autocorrelation with constant variance across trial blocks and subjects; ${\sigma }_{{e}_{i}}^{2}{\mathbf{I}}_{9}$ denotes independence between trials, homogeneity of variance across trial blocks and heterogeneity of variance between subjects; $AR(1)$ with ${\sigma }_{{e}_{i}}^{2}$ denotes a first-order autocorrelation with constant variance across trial blocks, homogeneity of the autocorrelation between subjects, and heterogeneity of variance between subjects

We first examined the overall impact of including the autocorrelation residual structure in the mixed-effects models (Models 3a and 3b) and the mixed-effects location scale models (Models 4a and 4b). Models 3a and 4a assumed independence between the residuals and Models 3b and 4b assume an AR(1) structure. A deviance test comparing Models 3a and 3b was significant^4^, suggesting that dependencies in the scores were not entirely accounted for by the logistic growth function. The estimated autocorrelation under Model 3b was .39. Model 3b is also preferred to Model 3a according to the lower AIC and BIC indices under Model 3b. A significant deviance test comparing Models 4a and 4b (χ^2^(1 *df*) = 27.8, *p* < .001) and lower AIC and BIC values under Model 4b suggest that dependencies in the scores were not fully accounted for by the growth function. The estimated autocorrelation under Model 4b was .44. Thus, whether one is fitting a mixed-effects model or a mixed-effects location scale model, it can be important to test the assumption of independence between residuals at the first level of a model.

Allowing for autocorrelation between the trial-level residuals impacted the estimated variances of the random effects at the subject level when comparing the mixed-effects models, as also documented elsewhere (e.g., Chi & Reinsel, 1989; Blozis & Harring, 2021). Under Models 3a and 3b, the estimated variance of the random intercept was 91.7 under Model 3a and increased to 137 under Model 3b. The variance of the random asymptote was 68.0 under Model 3a and decreased to 61.5 under Model 3b. The variance of the random rate parameter was 0.10 under both Models 3a and 3b. Despite differences in the estimated effects of MK and CS on the coefficients of the growth model, the general interpretations are similar.

Relative to Models 3a and 3b, Models 4a and 4b were far more complex because they permitted heterogeneity of the covariance structure at both levels of the model. We first examined the estimates of the level 1 covariance structure for Models 4a and 4b. The estimated residual variance when both MK and CS were equal to their respective sample means was 7.9 under Model 4a and increased to 12.7 under Model 4b. The estimated effect of CS on the residual variance was close to 0 and not statistically significant under both Models 4a and 4b, and the effect of MK, also small in both models, was only significant under Model 4a. The most notable consequence of ignoring autocorrelation at the trial level was apparent when examining the point estimate of the variance of the random scale effect under Model 4a that assumed independence and Model 4b that assumed an AR(1) structure. The estimated variance of the random scale effect was reduced from 0.51 under Model 4a to 0.32 under Model 4b. Similar to the first example presented in this paper, this result suggests that between-subject heterogeneity of the residual variance was due in part to dependencies between the residuals after accounting for growth by the subject-specific model.

Unlike Models 3a and 3b that assumed homogeneity of the covariance structure at the subject level, Models 4a and 4b specified that the variances of the conditional random growth coefficients to be functions of the measured covariates MK and CS, and consequently, the variances of the conditional random growth coefficients are the variances when both MK and CS are at their respective sample means. Given that the effects of MK and CS are significant, it is natural to expect that the estimated variances of the conditional coefficients under Models 4a or 4b to differ from the estimates obtained under Models 3a or 3b, and indeed, the estimates do (see Table 2). Thus, we turn to study the impact of ignoring the autocorrelation in the trial-level residuals when interpreting the subject-level covariance structure.

We examined both the estimated variances of the conditional random growth coefficients and the effects of MK and CS on these variances. The estimated variance of the conditional random intercept was lower when the trial-level residuals are allowed to correlate: The estimated variance was 89.7 under Model 4a and was reduced to 65.7 under Model 4b. The estimated effects of MK and CS on this variance were also impacted by the trial-level covariance structure: The estimated effect of MK increased (in absolute value) from –0.08 under Model 4a to –0.12 under Model 4b; although slight, the estimated effect of MK increased from 0.02 under Model 4a to 0.03 under Model 4b. The degree of precision of the estimates decreased, however, when the trial-level residuals were allowed to correlate, as the estimated confidence intervals for the effects increased under Model 4b. Next, the estimated variance of the conditional asymptote was lower when the trial-level residuals were allowed to correlate: The estimated variance was 62.3 under Model 4a and 47.1 under Model 4b. Although the estimated effects of MK and CS differed between Models 4a and 4b, neither were statistically significant under either model. Finally, the estimated variance of the conditional rate parameter was lower when the trial-level residuals were allowed to correlate: The estimated variance was 0.12 under Model 4a and 0.06 under Model 4b. The estimated effects of MK and CS also differed between Models 4a and 4b: the magnitude of the effect of MK increased from –0.15 under Model 4a to –0.25 under Model 4b, and although the estimated effect of CS differed between models, the effect was not statistically significant under either model. We again note the trade-off in fitting the more complex model by the decrease in the precision of the estimates evidenced by wider estimated confidence intervals.

### Example 3: Performance on a quantitative skill acquisition task

The third data set involves response latencies on a procedural learning task designed to measure quantitative skill acquisition^5^. Participants were instructed to learn a set of declarative rules for evaluating characteristics of visual stimuli that were presented in a series of trials. Each of the 12 scores represents the median time to respond across a block of 32 trials. Scores for the first trial block are not analyzed, assuming that participants were adapting to the task, leaving data for trials 2–12 for analysis. Included in a battery of individual difference measures was one of working-memory capacity obtained using a quantitative verification span paradigm. The quantitative working-memory capacity measure (QWM) is used to test how working memory capacity is related to learning acquisition.

**Fig. 3:**
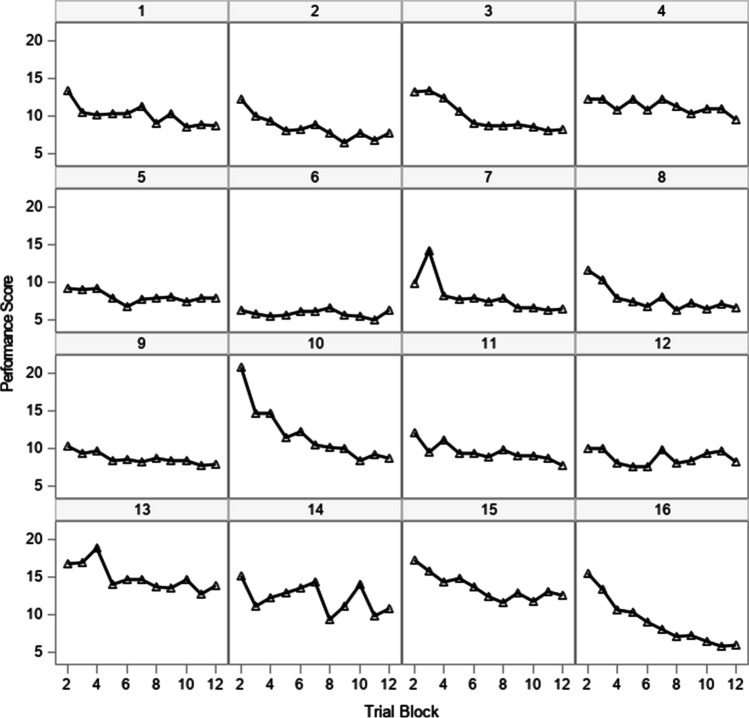
Response latencies on a quantitative skill acquisition task for a selection of 16 participants

Descriptive statistics for the response latencies and working-memory capacity are in Supplemental Table S3. Scores for 16 selected participants are displayed in Fig. 3. These scores were analyzed by applying a negatively accelerated exponential function, and like the preceding examples, alternative covariance structures are applied to understand the different sources of variation in performance scores, with working-memory capacity included in the models to test its relation to different aspects of learning and sources of score variation. We used the third data set to specifically focus on the use of an AR(1) structure as a means for reducing the dimensionality of nonlinear mixed-effects and nonlinear mixed-effects location scale models. As the dimensionality of a mixed-effects model increases, so does the computational demands. Given the relative complexity of a mixed-effects location scale model to a mixed-effects model, researchers might consider this strategy to help improve the computational demands of fitting these models.

To begin, we applied three nonlinear mixed-effects models based on different growth functions to the performance scores. Among them, the exponential function^6^ in (2), with each random coefficient specified as a function of QWM, provided the best fit:

11a $${\beta }_{0i}={\gamma }_{00}+{\gamma }_{01}QW{M}_{i}+{u}_{0i},$$

11b $${\beta }_{1i}={\gamma }_{10}+{\gamma }_{11}{QWM}_{i}+{u}_{1i},$$

11c $${\beta }_{2i}={\gamma }_{20}+{\gamma }_{21}{QWM}_{i}+{u}_{2i},$$

where ${\gamma }_{00}$, ${\gamma }_{10}$, and ${\gamma }_{20}$ are the performance levels at the first trial, potential level, and rate parameter, respectively, for a subject with QWM equal to the sample mean and whose random effects ${u}_{0i}$, ${u}_{1i}$, and ${u}_{2i}$ are equal to 0. The coefficients ${\gamma }_{01}$, ${\gamma }_{11}$, and ${\gamma }_{21}$ are the effects of QWM on the three learning coefficients. The residuals of (11a)–(11c), ${u}_{0i}$, ${u}_{1i}$, and ${u}_{2i}$, are the subject-specific effects after accounting for the effects of QWM. This model, Model 5a, assumed that the trial-level residuals were i.i.d. normal and independent with constant variance across trials and subjects: ${{\boldsymbol{\Theta}}}_{\varepsilon }={\sigma }_{\varepsilon }^{2}{\mathbf{I}}_{11}$, where ${\sigma }_{\varepsilon }^{2}$ is the common variance.

Next, we fitted Model 5b that assumed a fixed rate parameter to test whether a random rate parameter was needed. A deviance test between these models suggested that the rate parameter varied between subjects (χ^2^(3 *df*) = 39, *p* < .001). To then explore the idea of using an AR(1) structure to reduce the number of random effects, a third model, Model 5c, was fit that assumed an AR(1) structure at level 1 and a fixed nonlinear rate parameter. We then compared the fit of Model 5a that assumed all three growth coefficients were random and that the level-1 residuals were independent between trials to the fit of Model 5c that assumed two random growth coefficients (intercept and asymptote), a fixed rate parameter, and an AR(1) structure at the trial level. According to the AIC and BIC, model fit was relatively better under Model 5c, suggesting that an AR(1) structure might be used to reduce the number of random coefficients in a growth model, and consequently the total number of model parameters and computational demands, while achieving better overall model fit. The mean and covariance structures of Models 5a–5c are summarized in Supplemental Table S4. Estimates and fit indices for Models 5a–5c are in Table 3.

**Table 3:** ML estimates of a mixed-effects model for quantitative skill acquisition scores (n = 204)

	Model 5a	Model 5b	Model 5c
Within-subject covariance structure	${\sigma }_{e}^{2}{\mathbf{I}}_{11}$	${\sigma }_{e}^{2}{\mathbf{I}}_{11}$	$AR(1)$ with ${\sigma }_{e}^{2}$
Fixed effects	MLE [95% CI]	MLE [95% CI]	MLE [95% CI]
Initial level, ${\gamma }_{10}$	2.46 [2.44, 2.48]	2.45 [2.42, 2.48]	2.47 [2.45, 2.50]
QWM, ${\gamma }_{11}$	−0.30 [−0.39, −0.21]	−0.30 [−0.46, −0.14]	−0.34 [−0.46, −0.21]
Asymptote, ${\gamma }_{20}$	2.00 [1.98, 2.03]	2.05 [2.02, 2.08]	2.06 [2.03, 2.09]
QWM, ${\gamma }_{21}$	−0.24 [−0.31, −0.17]	−0.30 [−0.40, −0.20]	−0.28 [−0.40, −0.16]
Rate, ${\gamma }_{30}$	0.26 [0.23, 0.30]	0.24 [0.22, 0.27]	0.25 [0.22, 0.28]
QWM, ${\gamma }_{31}$	−0.07 [−0.13, −0.01]	−0.19 [−0.30, −0.08]	−0.17 [−0.30, −0.05]
Within-subject covariance parameters			
${\tau }_{0}$	−5.0 [−5.0, −4.9]	−4.9 [−5.0, −4.8]	−4.8 [−4.9, −4.7]
$\rho$			.22
Between-subject covariance parameters			
Intercept, ${\alpha }_{10}$	−2.5 [−2.6, −2.3]	−2.5 [−2.6, −2.3]	−2.4 [−2.6, −2.2]
Asymptote, ${\alpha }_{20}$	−3.1 [−3.3 , −3.0]	−2.9 [−3.1, −2.7]	−2.9 [−3.1, −2.7]
Rate, ${\alpha }_{30}$	−4.1 [−4.4, − 3.8]		
Corr(${u}_{2},{u}_{1}$), ${\phi }_{{u}_{2}{u}_{1}}$	.65	.72	.76
Corr(${u}_{3},{u}_{1}$), ${\phi }_{{u}_{3}{u}_{1}}$	−.16		
Corr(${u}_{3},{u}_{2}$), ${\phi }_{{u}_{3}{u}_{2}}$	.09		
Additional variance estimates			
Residual, ${\sigma }_{{e}_{0}}^{2}$	0.007	0.007	0.008
Initial, ${\phi }_{{u}_{1}}^{2}$	0.085	0.097	0.090
Asymptote, ${\phi }_{{u}_{2}}^{2}$	0.044	0.058	0.054
Rate, ${\phi }_{{u}_{3}}^{2}$	0.017		
−2lnL	−3421	−3382	−3430
AIC	−3395	−3362	−3408
BIC	−3352	−3329	−3372

QWM = Quantitative working-memory capacity (centered to the sample mean). The within-subject covariance structures are as follows: ${\sigma }_{e}^{2}{\mathbf{I}}_{11}$ denotes independence between trials and homogeneity of variance across trial blocks and subjects; $AR(1)$ with ${\sigma }_{e}^{2}$ denotes a first-order autocorrelation with constant variance across trial blocks and subjects; ${\sigma }_{{e}_{i}}^{2}{\mathbf{I}}_{11}$ denotes independence between trials, homogeneity of variance across trial blocks and heterogeneity of variance between subjects; $AR(1)$ with ${\sigma }_{{e}_{i}}^{2}$ denotes a first-order autocorrelation with constant variance across trial blocks, homogeneity of the autocorrelation between subjects, and heterogeneity of variance between subjects

Next, we followed a similar strategy in fitting models in the context of a mixed-effects location scale model. The first model, Model 6a, was a mixed-effects location scale model in which the three growth coefficients were random, as in Model 5a; the level-1 residuals were assumed to be independent with constant variance across trials, but the variance could vary by subject according to QWM and unmeasured sources: ${{\boldsymbol{\Theta}}}_{{\varepsilon }_{ij}}={\sigma }_{{\varepsilon }_{i}}^{2}{\mathbf{I}}_{11}$, where

$${\sigma }_{{\varepsilon }_{i}}^{2}=exp\left({\tau }_{0}+{{\tau }_{1}{QWM}_{i}+v}_{i}\right),$$

where ${\tau }_{0}$, when exponentiated, is the residual variance for a subject whose QWM score is equal to the sample mean and random scale effect ${v}_{i}$ is equal to 0; ${\tau }_{1}$ is the effect of QWM on the exponent. The random scale effect ${v}_{i}$ is the residual after accounting for QWM and is assumed to be lognormally and independently distributed between subjects. Adding a random scale effect, the level 2 covariance matrix included the variance of the random scale and its covariances with the conditional random effects of the growth model, like (7).

A second model, Model 6b, assumed a fixed rate parameter so that we could test whether a random rate parameter was needed under this mixed-effects location scale model. A deviance test between Models 6a and 6b suggested that the rate parameter varied by subject (χ^2^(3 *df*) = 30, *p* < .001). We then reduced the complexity of the growth model by fixing the rate parameter and adding an AR(1) structure to create a third model, Model 6c, where it was assumed that the autocorrelation coefficient was fixed and the residual variance was random. We then compared the fit of Model 6a that assumed all three growth coefficients were random and the level-1 residuals were independent between trials to the fit of Model 6c that assumed two random growth coefficients (intercept and asymptote), a fixed rate parameter and an AR(1) structure. According to the AIC and BIC values, model fit was better under Model 6c, suggesting that an AR(1) structure could be used to reduce the number of random coefficients in a mixed-effects location scale model while improving model fit. Estimates, 95% confidence intervals, and indices of model fit for Models 6a–6c are in Table 4. The mean and covariance structures of Models 6a–6d are summarized in Supplemental Table S4.

**Table 4:** ML estimates of a mixed-effects location scale model for quantitative skill acquisition scores (n = 204)

	Model 6a	Model 6b	Model 6c
Within-subject covariance structure	${\sigma }_{{e}_{i}}^{2}{\mathbf{I}}_{11}$	${\sigma }_{{e}_{i}}^{2}{\mathbf{I}}_{11}$	$AR(1)$ with ${\sigma }_{{e}_{i}}^{2}$
Fixed effects	MLE [95% CI]	MLE [95% CI]	MLE [95% CI]
Initial level, ${\gamma }_{10}$	2.46 [2.43, 2.49]	2.48 [2.46, 2.49]	2.48 [2.46, 2.51]
QWM, ${\gamma }_{11}$	−0.29 [−0.38, −0.20]	−0.30 [−0.39, −0.21]	−0.29 [−0.42, −0.16]
Asymptote, ${\gamma }_{20}$	2.02 [1.99, 2.05]	2.08 [2.06, 2.10]	2.08 [2.05, 2.10]
QWM, ${\gamma }_{21}$	−0.35 [−0.43, −0.28]	−0.24 [−0.31, −0.17]	−0.24 [−0.34, −0.13]
Rate, ${\gamma }_{30}$	0.26 [0.22, 0.29]	0.26 [0.24, 0.29]	0.27 [0.24, 0.30]
QWM, ${\gamma }_{31}$	−0.20 [−0.31, −0.10]	−0.14 [−0.24, −0.05]	−0.16 [−0.27, −0.04]
Within-subject covariance parameters			
${\tau }_{0}$	−5.1 [−5.3, −5.0]	−5.1 [−5.2, −5.0]	−5.0 [−5.1, −4.9]
QWM, ${\tau }_{1}$	−0.48 [−1.0, 0.06]	−0.34 [−0.84, 0.16]	−0.38 [−0.89, 0.14]
$\rho$			.22
Between-subject covariance parameters			
Intercept, ${\alpha }_{10}$	−2.7 [−2.9, −2.6]	−2.7 [−2.7, −2.6]	−2.6 [−2.8, −2.5]
QWM, ${\alpha }_{11}$	−0.67 [−1.1, −0.24]	−0.78 [−1.2, −0.37]	−0.64 [−1.2, −0.04]
Asymptote, ${\alpha }_{20}$	−3.4 [−3.7, −3.2]	−3.0 [−3.1, −2.9]	−3.0 [−3.1, −2.9]
QWM, ${\alpha }_{21}$	−0.02 [−0.68, 0.63]	−0.18 [−0.61, 0.25]	−0.17 [−0.83, 0.48]
Rate, ${\alpha }_{30}$	−4.4 [−4.7, −4.2]		
QWM, ${\alpha }_{31}$	0.71 [−0.50, 1.9]		
Scale, ${\alpha }_{v}$	−0.47 [−0.63, −0.33]	−0.49 [−0.66, −0.35]	−0.48 [−0.64, −0.33]
Corr(${u}_{2},{u}_{1}$), ${\phi }_{{u}_{2}{u}_{1}}$	.64	.67	.69
Corr(${u}_{3},{u}_{1}$), ${\phi }_{{u}_{3}{u}_{1}}$	−.23		
Corr(${u}_{3},{u}_{2}$), ${\phi }_{{u}_{3}{u}_{2}}$	.02		
Corr(*v* $,{u}_{1}$), ${\phi }_{v{u}_{1}}$	.36	.42	.43
Corr(*v* $,{u}_{2}$), ${\phi }_{v{u}_{2}}$	.45	.56	.58
Corr(*v* $,{u}_{3}$), ${\phi }_{v{u}_{3}}$	−.35		
Additional variance estimates			
Residual, ${\sigma }_{{e}_{0}}^{2}$	0.006	0.006	0.007
Initial, ${\phi }_{{u}_{1}}^{2}$	0.064	0.070	0.071
Asymptote, ${\phi }_{{u}_{2}}^{2}$	0.032	0.051	0.049
Rate, ${\phi }_{{u}_{3}}^{2}$	0.012		
Scale, ${\phi }_{v}^{2}$	0.39	0.37	0.39
−2lnL	−3613	−3583	−3637
AIC	−3571	−3551	−3603
BIC	−3501	−3498	−3546

QWM = Quantitative working-memory capacity (centered to the sample mean). The within-subject covariance structures are as follows: ${\sigma }_{e}^{2}{\mathbf{I}}_{11}$ denotes independence between trials and homogeneity of variance across trial blocks and subjects; $AR(1)$ with ${\sigma }_{e}^{2}$ denotes a first-order autocorrelation with constant variance across trial blocks and subjects; ${\sigma }_{{e}_{i}}^{2}{\mathbf{I}}_{11}$ denotes independence between trials, homogeneity of variance across trial blocks and heterogeneity of variance between subjects; $AR(1)$ with ${\sigma }_{{e}_{i}}^{2}$ denotes a first-order autocorrelation with constant variance across trial blocks, homogeneity of the autocorrelation between subjects, and heterogeneity of variance between subjects

## Discussion

The collection of repeated measures and longitudinal data is central for investigations that seek to understand change, development, or growth in measured behaviors. Mixed-effects models, a popular choice in statistical methodology, have evolved considerably since they were introduced in Laird and Ware (1982). The advantages of this major statistical framework that unify models for the population-level response and that of the individual are now numerous, including, but not limited to, the specification of linear and nonlinear models and a wide range of response distributions, handling of missing data, and data observed at different times for different subjects.

In applying a mixed-effects model to data, it is important to consider the structures necessary to address sources of heterogeneity of variance, both within and between subjects. This may be done to improve statistical inference or model fit (Blozis & Harring, 2021; Chi & Reinsel, 1989; Ferron et al., 2002; Funatogawa & Funatogawa, 2018; Harring & Blozis, 2014; Sivo et al., 2005). For instance, Chi and Reinsel show that neglecting serial correlation between level-1 residuals can result in overestimation of the variances of the random effects at the second level of a linear mixed-effects model, and Blozis and Harring showed how assumptions about the residual covariance structure (including serial correlations and heterogeneity of variance) can impact the estimated variances of the random effects at the subject level of a nonlinear mixed-effects model. Recommendations relating to statistical inference about the random-effects covariance structure at the subject level are that researchers consider alternative residual covariance structures at the first level. This is especially relevant given recent developments in mixed-effects location scale models that specifically aim to model heterogeneity of variance at both levels (Blozis et al., 2020; Hedeker & Nordgren, 2013; Williams et al., 2019).

Considering computational burden, we also considered a point of discussion in Chi and Reinsel (1989) that concerned the application of linear mixed-effects models to repeated measures data. Specifically, they discuss the use of an AR(1) structure for the level-1 residuals as a potential means to reduce the number of random effects needed to characterize a response. We explored this when fitting the nonlinear models here, including the nonlinear mixed-effects location scale models, as reducing the number of random coefficients could be helpful in reducing computational demands when fitting such complex models. Although this strategy was useful in the examples presented here, it is important to note that because one model provides a better fit than another, this is not to suggest that the best fitting model is the one that generated the data. It is simply that one might consider an alternative way of accounting for dependencies in the data. In the end, it is the individual researcher who makes the decision about how a model is to be specified to test particular questions about a behavior. The models presented here offer alternative methods for specifying models to test or account for heterogeneity of responses.

Estimation of mixed-effects models can be carried out using ML and Bayesian approaches. Although the current paper relies on ML, there are advantages to considering a Bayesian approach (Lin et al., 2018). For example, in one form of the model considered here, an AR(1) structure was applied to repeated measures data that permitted between-subject heterogeneity of the residual variance and the autocorrelation coefficient (see Example 2). The residual variance was modeled using an exponential function, and tests of covariate effects were carried out assuming that the effects were lognormal. Thus, this did not require special attention to the distributional assumptions made about the coefficients of the variance model. Estimation of this model using PROC NLMIXED, however, assumed that the random effect corresponding to the autocorrelation coefficient was approximated by a truncated normal distribution (with lower and upper bounds to limit the distribution between -1 and 1). Using a Bayesian approach, such as by using the SAS PROC MCMC statistical software program (Chen, 2009) would increase flexibility in the assumptions made about random effects.

### Electronic supplementary material

Below is the link to the electronic supplementary material.

Supplementary file1 (DOCX 15 KB)

Supplementary file2 (DOCX 25 KB)

## Appendix

/* SAS PROC NLMIXED is a procedure that can be used to estimate nonlinear mixed-effects models. For a two-level model, the default specification for the residual covariance structure at the first level is that the residuals are independent within and between level-2 units. The GENERAL model statement is used to specify alternative residual covariance structures.

Below is syntax for fitting a logistic growth model to a set of performance scores from the flight simulation task (the second example presented in the paper). The data set includes 9 repeated measures for each of 140 subjects. Two individual difference covariates, Mathematics Knowledge (MK) and Coding Speed (CS), are included in the models; each is centered about their respective sample mean. The within-subject residuals are assumed to be normally distributed.

Each script corresponds to the models reported in the manuscript for the second example. */

## Footnotes

1 Singer and Willett (2003) recommend using the level 1 sample size in calculating the BIC = −2loglikelihood + ln(N)k, where *N* is the number of units at level 1.

2 Here and where other deviance tests for a nonzero variance were carried out, a 50:50 mixture of ${\chi }_{q}^{2}$ and ${\chi }_{q+1}^{2}$, where *q* is the number of random effects at the subject level, was used to calculate the *p*-value (Snijders & Bosker, 2012).

3 MK and CS were centered about their respective sample means.

4 ($${\chi }^{2}$$ = 16.4, *p* = .5*.004

5 The data were provided by Scott Chaiken of the Armstrong Laboratory, Brooks Air Force Base.

6 Based on AIC index values, the exponential function in Eq. (2) yielded better fit to the data overall relative to a logistic function (Eq. 7) and a Gompertz function: ${y}_{\mathrm{ij}}={\beta }_{1i}exp\left\{ln\left({~}^{{\beta }_{2i}}\!\left/ \!{~}_{{\beta }_{1i}}\right.\right)exp\left\{-t{\beta }_{3i}\right\}\right\}+{\varepsilon }_{\mathrm{ij}}$.
