# Supplementary material for: Alternative covariance structures in mixed-effects models: Addressing intra- and inter-individual heterogeneity
Source: Behav Res Methods. 2023 May 25;56(3):2013–32. doi: 10.3758/s13428-023-02133-1 (PMC11327215; doi:10.3758/s13428-023-02133-1)
Supplement: Supplementary file 1 — Supplementary file1 (DOCX 15 KB) [file 13428_2023_2133_MOESM1_ESM.docx]

Supplemental Table S1

Descriptive Statistics for Response Latencies on a Procedural Learning Task (n = 393)

| Trial | Mean | SD | Median | SIQR | Minimum | Maximum |
| --- | --- | --- | --- | --- | --- | --- |
| 2 | 19.7 | 6.1 | 18.2 | 7.8 | 6.8 | 46.7 |
| 3 | 17.2 | 5.0 | 15.8 | 6.1 | 7.6 | 36.9 |
| 4 | 15.3 | 3.9 | 14.4 | 4.8 | 7.9 | 28.8 |
| 5 | 14.5 | 3.5 | 13.8 | 4.4 | 8.7 | 29.5 |
| 6 | 13.6 | 3.1 | 13.0 | 3.9 | 7.9 | 25.9 |
| 7 | 13.2 | 2.8 | 12.5 | 3.5 | 7.4 | 23.3 |
| 8 | 12.7 | 2.6 | 12.4 | 3.7 | 7.0 | 23.9 |
| 9 | 12.5 | 2.5 | 12.1 | 3.1 | 8.0 | 20.6 |
| 10 | 12.1 | 2.5 | 11.6 | 3.0 | 6.6 | 21.5 |
| 11 | 12.0 | 2.3 | 11.5 | 2.9 | 7.5 | 21.2 |

*Note:* SIQR is the semi-interquartile range.

Supplemental Table S2

Descriptive Statistics for Flight Simulation Performances Scores (n = 140)

|  |  |  | |  |  |  |  |
| --- | --- | --- | --- | --- | --- | --- | --- |
| Trial | Mean | SD | Median | | SIQR | Minimum | Maximum |
| 2 | 20.1 | 9.7 | 20.5 | | 7 | 0 | 42 |
| 3 | 25.5 | 9.7 | 26 | | 5.5 | 0 | 44 |
| 4 | 29.3 | 9.8 | 30 | | 6.3 | 0 | 46 |
| 5 | 32.4 | 9.3 | 33 | | 5.3 | 0 | 51 |
| 6 | 34.2 | 8.6 | 34 | | 5 | 2 | 52 |
| 7 | 35.6 | 7.7 | 35.5 | | 5 | 11 | 52 |
| 8 | 37.7 | 7.4 | 37.5 | | 5.5 | 21 | 60 |
| 9 | 39.0 | 7.8 | 38 | | 5.8 | 10 | 56 |
| 10 | 38.8 | 8.2 | 38 | | 5.3 | 10 | 56 |
| Individual Difference Measures | | |  | |  |  |  |
| MK | 17.2 | 4.6 | 17.0 | | 25 | 6 | 25 |
| CS | 55.9 | 12.1 | 54.5 | | 84 | 30 | 84 |

*Note:* SIQR is the semi-interquartile range.

Supplemental Table S3

Descriptive Statistics for Response Latencies on a Quantitative Skill Acquisition Task (n = 204)

|  |  |  | |  |  |  |  |
| --- | --- | --- | --- | --- | --- | --- | --- |
| Trial | Mean | SD | Median | | SIQR | Minimum | Maximum |
| 2 | 12.3 | 3.9 | 11.5 | | 2.4 | 5.8 | 27.4 |
| 3 | 10.9 | 3.3 | 10.0 | | 2.2 | 5.7 | 23.3 |
| 4 | 10.2 | 2.9 | 10.0 | | 2.1 | 5.4 | 20.8 |
| 5 | 9.7 | 2.7 | 9.0 | | 1.8 | 5.0 | 19.4 |
| 6 | 9.4 | 2.5 | 9.1 | | 1.7 | 4.9 | 18.0 |
| 7 | 9.1 | 2.4 | 8.7 | | 1.7 | 4.7 | 19.9 |
| 8 | 8.8 | 2.4 | 8.3 | | 1.5 | 4.9 | 18.3 |
| 9 | 8.6 | 2.2 | 8.2 | | 1.5 | 4.9 | 18.7 |
| 10 | 8.6 | 2.3 | 8.2 | | 1.5 | 4.7 | 18.5 |
| 11 | 8.4 | 2.1 | 8.0 | | 1.3 | 4.8 | 15.9 |
| 12 | 8.1 | 2.0 | 7.9 | | 1.4 | 4.6 | 14.7 |
| Individual Difference Measure | |  |  | |  |  |  |
| QWM | 78.0 | 19.3 | 81.9 | | 11.1 | 0.4 | 100 |

*Note:* SIQR is the semi-interquartile range.
