# Supplementary material for: Alternative covariance structures in mixed-effects models: Addressing intra- and inter-individual heterogeneity
Source: Behav Res Methods. 2023 May 25;56(3):2013–32. doi: 10.3758/s13428-023-02133-1 (PMC11327215; doi:10.3758/s13428-023-02133-1)
Supplement: Supplementary file 2 — Supplementary file2 (DOCX 25 KB) [file 13428_2023_2133_MOESM2_ESM.docx]

**Supplemental Table S4.**

*Mean and covariance structures of the models fitted to the performance data in three learning experiments.*

| Example 1: Performance on a Complex Procedural Learning Task (Woltz, 1988) | | | |
| --- | --- | --- | --- |
| Model | Mean structure | Level 1 Covariance Structure | Level 2 Covariance Structure |
| 1a | $E\left[ y_{\mathrm{ij}} \right]=\beta_{1}-\left( \beta_{1}-\beta_{0} \right)exp\left\{ -\beta_{2}t_{j} \right\}$ | $\boldsymbol{\Theta}_{\varepsilon}=\sigma_{\varepsilon}^{2}\mathbf{I}_{10}$ | $\boldsymbol{\Phi}=\left[ \begin{matrix} \phi_{u_{0}}^{2} & & \\ \phi_{u_{1}u_{0}} & \phi_{u_{1}}^{2} & \\ \phi_{u_{2}u_{0}} & \phi_{u_{2}u_{1}} & \phi_{u_{2}}^{2} \end{matrix} \right]$ |
| 1b | $E\left[ y_{\mathrm{ij}} \right]=\beta_{1}-\left( \beta_{1}-\beta_{0} \right)exp\left\{ -\beta_{2}t_{j} \right\}$ | $\boldsymbol{\Theta}_{\varepsilon}=\sigma_{\varepsilon}^{2}\left[ \begin{matrix} 1 & & & & \\ \rho& 1 & & & \\ \rho^{2} & \rho& 1 & & \\ \vdots& \vdots& \vdots& \ddots& \\ \rho^{9} & \rho^{8} & \rho^{7} & \cdots& 1 \end{matrix} \right]$ | $\boldsymbol{\Phi}=\left[ \begin{matrix} \phi_{u_{0}}^{2} & & \\ \phi_{u_{1}u_{0}} & \phi_{u_{1}}^{2} & \\ \phi_{u_{2}u_{0}} & \phi_{u_{2}u_{1}} & \phi_{u_{2}}^{2} \end{matrix} \right]$ |
| 1c | $E\left[ y_{\mathrm{ij}} \right]=\beta_{1}-\left( \beta_{1}-\beta_{0} \right)exp\left\{ -\beta_{2}t_{j} \right\}$ | $\boldsymbol{\Theta}_{\varepsilon}=\sigma_{\varepsilon}^{2}\left[ \begin{matrix} 1 & & & & \\ \rho& 1 & & & \\ \rho^{2} & \rho& 1 & & \\ \vdots& \vdots& \vdots& \ddots& \\ \rho^{9} & \rho^{8} & \rho^{7} & \cdots& 1 \end{matrix} \right]$ | $\boldsymbol{\Phi}=\left[ \begin{matrix} \phi_{u_{0}}^{2} & \\ \phi_{u_{1}u_{0}} & \phi_{u_{1}}^{2} \end{matrix} \right]$ |
| 2a | $E\left[ y_{\mathrm{ij}} \right]=\beta_{1}-\left( \beta_{1}-\beta_{0} \right)exp\left\{ -\beta_{2}t_{j} \right\}$ | $\boldsymbol{\Theta}_{\varepsilon_{i}}=\sigma_{\varepsilon_{i}}^{2}\mathbf{I}_{10}$  where $\sigma_{\varepsilon_{i}}^{2}=exp\left( \tau_{0}{+v}_{i} \right)$ | $\boldsymbol{\Phi}=\left[ \begin{matrix} \phi_{u_{0}}^{2} & & & \\ \phi_{u_{1}u_{0}} & \phi_{u_{1}}^{2} & & \\ \phi_{u_{2}u_{0}} & \phi_{u_{2}u_{1}} & \phi_{u_{2}}^{2} & \\ \phi_{{vu}_{0}} & \phi_{vu_{1}} & \phi_{vu_{2}} & \phi_{v}^{2} \end{matrix} \right]$ |
| 2b | $E\left[ y_{\mathrm{ij}} \right]=\beta_{1}-\left( \beta_{1}-\beta_{0} \right)exp\left\{ -\beta_{2}t_{j} \right\}$ | $\boldsymbol{\Theta}_{\varepsilon_{ij}}=\sigma_{\varepsilon_{i}}^{2}\left[ \begin{matrix} 1 & & & & \\ \rho& 1 & & & \\ \rho^{2} & \rho& 1 & & \\ \vdots& \vdots& \vdots& \ddots& \\ \rho^{9} & \rho^{8} & \rho^{7} & \cdots& 1 \end{matrix} \right]$  where $\sigma_{\varepsilon_{i}}^{2}=exp\left( \tau_{0}{+v}_{i} \right)$ | $\boldsymbol{\Phi}=\left[ \begin{matrix} \phi_{u_{0}}^{2} & & & \\ \phi_{u_{1}u_{0}} & \phi_{u_{1}}^{2} & & \\ \phi_{u_{2}u_{0}} & \phi_{u_{2}u_{1}} & \phi_{u_{2}}^{2} & \\ \phi_{{vu}_{0}} & \phi_{vu_{1}} & \phi_{vu_{2}} & \phi_{v}^{2} \end{matrix} \right]$ |
| 2c | $E\left[ y_{\mathrm{ij}} \right]=\beta_{1}-\left( \beta_{1}-\beta_{0} \right)exp\left\{ -\beta_{2}t_{j} \right\}$ | $\boldsymbol{\Theta}_{\varepsilon_{ij}}=\sigma_{\varepsilon_{i}}^{2}\left[ \begin{matrix} 1 & & & & \\ \rho& 1 & & & \\ \rho^{2} & \rho& 1 & & \\ \vdots& \vdots& \vdots& \ddots& \\ \rho^{9} & \rho^{8} & \rho^{7} & \cdots& 1 \end{matrix} \right]$  where $\sigma_{\varepsilon_{i}}^{2}=exp\left( \tau_{0}{+v}_{i} \right)$ | $\boldsymbol{\Phi}=\left[ \begin{matrix} \phi_{u_{0}}^{2} & & \\ \phi_{u_{1}u_{0}} & \phi_{u_{1}}^{2} & \\ \phi_{{vu}_{0}} & \phi_{vu_{1}} & \phi_{v}^{2} \end{matrix} \right]$ |
| Example 2: Performance on a Flight Controller Simulation Task | | | |
| 3a | $E\left[ y_{\mathrm{ij}} \right]=\frac{\beta_{0}\beta_{1}}{\beta_{0}+\left( \beta_{1}-\beta_{0} \right)exp\left\{ -\beta_{2}\left( t_{j}-1 \right) \right\}}$ | $\boldsymbol{\Theta}_{\varepsilon}=\sigma_{\varepsilon}^{2}\mathbf{I}_{9}$ | $\boldsymbol{\Phi}=\left[ \begin{matrix} \phi_{u_{0}}^{2} & & \\ \phi_{u_{1}u_{0}} & \phi_{u_{1}}^{2} & \\ \phi_{u_{2}u_{0}} & \phi_{u_{2}u_{1}} & \phi_{u_{2}}^{2} \end{matrix} \right]$ |
| 3b | $E\left[ y_{\mathrm{ij}} \right]=\frac{\beta_{0}\beta_{1}}{\beta_{0}+\left( \beta_{1}-\beta_{0} \right)exp\left\{ -\beta_{2}\left( t_{j}-1 \right) \right\}}$ | $\boldsymbol{\Theta}_{\varepsilon}=\sigma_{\varepsilon}^{2}\left[ \begin{matrix} 1 & & & & \\ \rho& 1 & & & \\ \rho^{2} & \rho& 1 & & \\ \vdots& \vdots& \vdots& \ddots& \\ \rho^{8} & \rho^{7} & \rho^{6} & \cdots& 1 \end{matrix} \right]$ | $\boldsymbol{\Phi}=\left[ \begin{matrix} \phi_{u_{0}}^{2} & & \\ \phi_{u_{1}u_{0}} & \phi_{u_{1}}^{2} & \\ \phi_{u_{2}u_{0}} & \phi_{u_{2}u_{1}} & \phi_{u_{2}}^{2} \end{matrix} \right]$ |
| 4a | $E\left[ y_{\mathrm{ij}} \right]=\frac{\beta_{0}\beta_{1}}{\beta_{0}+\left( \beta_{1}-\beta_{0} \right)exp\left\{ -\beta_{2}\left( t_{j}-1 \right) \right\}}$ | $\boldsymbol{\Theta}_{\varepsilon_{i}}=\sigma_{\varepsilon_{i}}^{2}\mathbf{I}_{9}$  where  $\sigma_{\varepsilon_{i}}^{2}=exp\left( \tau_{0}+{\tau_{1}{MK}_{i}+{\tau_{2}CS}_{i}+v}_{i} \right)$ | $\boldsymbol{\Phi}=\left[ \begin{matrix} \phi_{u_{0}}^{2} & & & \\ \phi_{u_{1}u_{0}} & \phi_{u_{1}}^{2} & & \\ \phi_{u_{2}u_{0}} & \phi_{u_{2}u_{1}} & \phi_{u_{2}}^{2} & \\ \phi_{{vu}_{0}} & \phi_{vu_{1}} & \phi_{vu_{2}} & \phi_{v}^{2} \end{matrix} \right]$  where  $\phi_{u_{0}}^{2}=exp\left\{ \alpha_{00}+\alpha_{01}{MK}_{i}+\alpha_{02}{CS}_{i} \right\}$  $\phi_{u_{1}}^{2}=exp\left\{ \alpha_{10}+\alpha_{11}{MK}_{i}+\alpha_{12}{CS}_{i} \right\}$  $\phi_{u_{2}}^{2}=exp\left\{ \alpha_{20}+\alpha_{21}{MK}_{i}+\alpha_{22}{CS}_{i} \right\}$ |
| 4b | $E\left[ y_{\mathrm{ij}} \right]=\frac{\beta_{0}\beta_{1}}{\beta_{0}+\left( \beta_{1}-\beta_{0} \right)exp\left\{ -\beta_{2}\left( t_{j}-1 \right) \right\}}$ | $\boldsymbol{\Theta}_{\varepsilon_{i}}=\sigma_{\varepsilon_{i}}^{2}\left[ \begin{matrix} 1 & & & & \\ \rho_{i} & 1 & & & \\ \rho_{i}^{2} & \rho_{i} & 1 & & \\ \vdots& \vdots& \vdots& \ddots& \\ \rho_{i}^{8} & \rho_{i}^{7} & \rho_{i}^{6} & \cdots& 1 \end{matrix} \right]$  where  $\sigma_{\varepsilon_{i}}^{2}=exp\left( \tau_{0}+{\tau_{1}{MK}_{i}+{\tau_{2}CS}_{i}+v}_{i} \right)$  $\rho_{i}=\rho+w_{i}$ | $\boldsymbol{\Phi}=\left[ \begin{matrix} \phi_{u_{0}}^{2} & & & & \\ \phi_{u_{1}u_{0}} & \phi_{u_{1}}^{2} & & & \\ \phi_{u_{2}u_{0}} & \phi_{u_{2}u_{1}} & \phi_{u_{2}}^{2} & & \\ \phi_{{vu}_{0}} & \phi_{vu_{1}} & \phi_{vu_{2}} & \phi_{v}^{2} & \\ \phi_{{wu}_{0}} & \phi_{{wu}_{1}} & \phi_{{wu}_{2}} & \phi_{wv} & \phi_{w}^{2} \end{matrix} \right]$  where  $\phi_{u_{0}}^{2}=exp\left\{ \alpha_{00}+\alpha_{01}{MK}_{i}+\alpha_{02}{CS}_{i} \right\}$  $\phi_{u_{1}}^{2}=exp\left\{ \alpha_{10}+\alpha_{11}{MK}_{i}+\alpha_{12}{CS}_{i} \right\}$  $\phi_{u_{2}}^{2}=exp\left\{ \alpha_{20}+\alpha_{21}{MK}_{i}+\alpha_{22}{CS}_{i} \right\}$ |
| 4c | $E\left[ y_{\mathrm{ij}} \right]=\frac{\beta_{0}\beta_{1}}{\beta_{0}+\left( \beta_{1}-\beta_{0} \right)exp\left\{ -\beta_{2}\left( t_{j}-1 \right) \right\}}$ | $\boldsymbol{\Theta}_{\varepsilon_{i}}=\sigma_{\varepsilon_{i}}^{2}\left[ \begin{matrix} 1 & & & & \\ \rho_{i} & 1 & & & \\ \rho_{i}^{2} & \rho_{i} & 1 & & \\ \vdots& \vdots& \vdots& \ddots& \\ \rho_{i}^{8} & \rho_{i}^{7} & \rho_{i}^{6} & \cdots& 1 \end{matrix} \right]$  where  $\sigma_{\varepsilon_{i}}^{2}=exp\left( \tau_{0}+{\tau_{1}{MK}_{i}+{\tau_{2}CS}_{i}+v}_{i} \right)$  $\rho_{i}=\rho+w_{i}$ | $\boldsymbol{\Phi}=\left[ \begin{matrix} \phi_{u_{0}}^{2} & & & \\ \phi_{u_{1}u_{0}} & \phi_{u_{1}}^{2} & & \\ \phi_{{vu}_{0}} & \phi_{vu_{1}} & \phi_{v}^{2} & \\ \phi_{{wu}_{0}} & \phi_{{wu}_{1}} & \phi_{wv} & \phi_{w}^{2} \end{matrix} \right]$  where  $\phi_{u_{0}}^{2}=exp\left\{ \alpha_{00}+\alpha_{01}{MK}_{i}+\alpha_{02}{CS}_{i} \right\}$  $\phi_{u_{1}}^{2}=exp\left\{ \alpha_{10}+\alpha_{11}{MK}_{i}+\alpha_{12}{CS}_{i} \right\}$ |
| Example 3: Performance on a Quantitative Skill Acquisition Task | | | |
| 5a | $E\left[ y_{\mathrm{ij}} \right]=\beta_{1}-\left( \beta_{1}-\beta_{0} \right)exp\left\{ -\beta_{2}t_{j} \right\}$ | $\boldsymbol{\Theta}_{\varepsilon}=\sigma_{\varepsilon}^{2}\mathbf{I}_{11}$ | $\boldsymbol{\Phi}=\left[ \begin{matrix} \phi_{u_{0}}^{2} & & \\ \phi_{u_{1}u_{0}} & \phi_{u_{1}}^{2} & \\ \phi_{{u_{2}u}_{0}} & \phi_{u_{2}u_{1}} & \phi_{u_{2}}^{2} \end{matrix} \right]$ |
| 5b | $E\left[ y_{\mathrm{ij}} \right]=\beta_{1}-\left( \beta_{1}-\beta_{0} \right)exp\left\{ -\beta_{2}t_{j} \right\}$ | $\boldsymbol{\Theta}_{\varepsilon}=\sigma_{\varepsilon}^{2}\mathbf{I}_{11}$ | $\boldsymbol{\Phi}=\left[ \begin{matrix} \phi_{u_{0}}^{2} & \\ \phi_{u_{1}u_{0}} & \phi_{u_{1}}^{2} \end{matrix} \right]$ |
| 5c | $E\left[ y_{\mathrm{ij}} \right]=\beta_{1}-\left( \beta_{1}-\beta_{0} \right)exp\left\{ -\beta_{2}t_{j} \right\}$ | $\boldsymbol{\Theta}_{\varepsilon}=\sigma_{\varepsilon}^{2}\left[ \begin{matrix} 1 & & & & \\ \rho& 1 & & & \\ \rho^{2} & \rho& 1 & & \\ \vdots& \vdots& \vdots& \ddots& \\ \rho^{10} & \rho^{9} & \rho^{8} & \cdots& 1 \end{matrix} \right]$ | $\boldsymbol{\Phi}=\left[ \begin{matrix} \phi_{u_{0}}^{2} & \\ \phi_{u_{1}u_{0}} & \phi_{u_{1}}^{2} \end{matrix} \right]$ |
| 6a | $E\left[ y_{\mathrm{ij}} \right]=\beta_{1}-\left( \beta_{1}-\beta_{0} \right)exp\left\{ -\beta_{2}t_{j} \right\}$ | $\boldsymbol{\Theta}_{\varepsilon_{i}}=\sigma_{\varepsilon_{i}}^{2}\mathbf{I}_{11}$  where  $\sigma_{\varepsilon_{i}}^{2}=exp\left( \tau_{0}+{\tau_{1}{QWM}_{i}+v}_{i} \right)$ | $\boldsymbol{\Phi}=\left[ \begin{matrix} \phi_{u_{0}}^{2} & & & \\ \phi_{u_{1}u_{0}} & \phi_{u_{1}}^{2} & & \\ \phi_{{u_{2}u}_{0}} & \phi_{u_{2}u_{1}} & \phi_{u_{2}}^{2} & \\ \phi_{{vu}_{0}} & \phi_{{vu}_{1}} & \phi_{{vu}_{2}} & \phi_{v}^{2} \end{matrix} \right]$  where  $\phi_{u_{0}}^{2}=exp\left\{ \alpha_{00}+\alpha_{01}{QWM}_{i} \right\}$  $\phi_{u_{1}}^{2}=exp\left\{ \alpha_{10}+\alpha_{11}{QWM}_{i} \right\}$  $\phi_{u_{2}}^{2}=exp\left\{ \alpha_{20}+\alpha_{21}{QWM}_{i} \right\}$ |
| 6b | $E\left[ y_{\mathrm{ij}} \right]=\beta_{1}-\left( \beta_{1}-\beta_{0} \right)exp\left\{ -\beta_{2}t_{j} \right\}$ | $\boldsymbol{\Theta}_{\varepsilon_{i}}=\sigma_{\varepsilon_{i}}^{2}\mathbf{I}_{11}$  where  $\sigma_{\varepsilon_{i}}^{2}=exp\left( \tau_{0}+{\tau_{1}{QWM}_{i}+v}_{i} \right)$ | $\boldsymbol{\Phi}=\left[ \begin{matrix} \phi_{u_{0}}^{2} & & \\ \phi_{u_{1}u_{0}} & \phi_{u_{1}}^{2} & \\ \phi_{{vu}_{0}} & \phi_{vu_{1}} & \phi_{v}^{2} \end{matrix} \right]$  where  $\phi_{u_{0}}^{2}=exp\left\{ \alpha_{00}+\alpha_{01}{QWM}_{i} \right\}$  $\phi_{u_{1}}^{2}=exp\left\{ \alpha_{10}+\alpha_{11}{QWM}_{i} \right\}$ |
| 6c | $E\left[ y_{\mathrm{ij}} \right]=\beta_{1}-\left( \beta_{1}-\beta_{0} \right)exp\left\{ -\beta_{2}t_{j} \right\}$ | $\boldsymbol{\Theta}_{\varepsilon_{i}}=\sigma_{\varepsilon_{i}}^{2}\left[ \begin{matrix} 1 & & & & \\ \rho& 1 & & & \\ \rho^{2} & \rho& 1 & & \\ \vdots& \vdots& \vdots& \ddots& \\ \rho^{10} & \rho^{9} & \rho^{8} & \cdots& 1 \end{matrix} \right]$  where  $\sigma_{\varepsilon_{i}}^{2}=exp\left( \tau_{0}+{\tau_{1}{QWM}_{i}+v}_{i} \right)$ | $\boldsymbol{\Phi}=\left[ \begin{matrix} \phi_{u_{0}}^{2} & & \\ \phi_{u_{1}u_{0}} & \phi_{u_{1}}^{2} & \\ \phi_{{vu}_{0}} & \phi_{vu_{1}} & \phi_{v}^{2} \end{matrix} \right]$  where  $\phi_{u_{0}}^{2}=exp\left\{ \alpha_{00}+\alpha_{01}{QWM}_{i} \right\}$  $\phi_{u_{1}}^{2}=exp\left\{ \alpha_{10}+\alpha_{11}{QWM}_{i} \right\}$ |
| 6d | $E\left[ y_{\mathrm{ij}} \right]=\beta_{1}-\left( \beta_{1}-\beta_{0} \right)exp\left\{ -\beta_{2}t_{j} \right\}$ | $\boldsymbol{\Theta}_{\varepsilon_{i}}=\sigma_{\varepsilon_{i}}^{2}\left[ \begin{matrix} 1 & & & & \\ \rho_{i} & 1 & & & \\ \rho_{i}^{2} & \rho_{i} & 1 & & \\ \vdots& \vdots& \vdots& \ddots& \\ \rho_{i}^{10} & \rho_{i}^{9} & \rho_{i}^{8} & \cdots& 1 \end{matrix} \right]$  where  $\sigma_{\varepsilon_{i}}^{2}=exp\left( \tau_{0}+{\tau_{1}{QWM}_{i}+v}_{i} \right)$  $\rho_{i}=\rho+w_{i}$ | $\boldsymbol{\Phi}=\left[ \begin{matrix} \phi_{u_{0}}^{2} & & & \\ \phi_{u_{1}u_{0}} & \phi_{u_{1}}^{2} & & \\ \phi_{{vu}_{0}} & \phi_{vu_{1}} & \phi_{v}^{2} & \\ \phi_{{wu}_{0}} & \phi_{{wu}_{1}} & \phi_{wv} & \phi_{w}^{2} \end{matrix} \right]$  where  $\phi_{u_{0}}^{2}=exp\left\{ \alpha_{00}+\alpha_{01}{QWM}_{i} \right\}$  $\phi_{u_{1}}^{2}=exp\left\{ \alpha_{10}+\alpha_{11}{QWM}_{i} \right\}$ |
